# Local and wide-scale livestock movement networks inform disease control strategies in East Africa

**DOI:** 10.1038/s41598-023-35968-x

**Published:** 2023-06-14

**Authors:** Divine Ekwem, Jessica Enright, J. Grant C. Hopcraft, Joram Buza, Gabriel Shirima, Mike Shand, James K. Mwajombe, Bernard Bett, Richard Reeve, Tiziana Lembo

**Affiliations:** 1 Boyd Orr Centre for Population and Ecosystem Health, School of Biodiversity, One Health & Veterinary Medicine, College of Medical, Veterinary & Life Sciences, University of Glasgow, Glasgow, UK; 2 School of Computing Science, University of Glasgow, Glasgow, UK; 3 The Nelson Mandela African Institution of Science and Technology, Arusha, Tanzania; 4 School of Geographical & Earth Sciences, University of Glasgow, Glasgow, UK; 5 Tanzania Agricultural Research Institute, Ministry of Agriculture, Arusha, Tanzania; 6 International Livestock Research Institute, Nairobi, Kenya

**Keywords:** Ecology, Environmental social sciences, Diseases, Risk factors

## Abstract

Livestock mobility exacerbates infectious disease risks across sub-Saharan Africa, but enables critical access to grazing and water resources, and trade. Identifying locations of high livestock traffic offers opportunities for targeted control. We focus on Tanzanian agropastoral and pastoral communities that account respectively for over 75% and 15% of livestock husbandry in eastern Africa. We construct networks of livestock connectivity based on participatory mapping data on herd movements reported by village livestock keepers as well as data from trading points to understand how seasonal availability of resources, land-use and trade influence the movements of livestock. In communities that practise agropastoralism, inter- and intra-village connectivity through communal livestock resources (e.g. pasture and water) was 1.9 times higher in the dry compared to the wet season suggesting greater livestock traffic and increased contact probability. In contrast, livestock from pastoral communities were 1.6 times more connected at communal locations during the wet season when they also tended to move farther (by 3 km compared to the dry season). Trade-linked movements were twice more likely from rural to urban locations. Urban locations were central to all networks, particularly those with potentially high onward movements, for example to abattoirs, livestock holding grounds, or other markets, including beyond national boundaries. We demonstrate how livestock movement information can be used to devise strategic interventions that target critical livestock aggregation points (i.e. locations of high centrality values) and times (i.e. prior to and after the wet season in pastoral and agropastoral areas, respectively). Such targeted interventions are a cost-effective approach to limit infection without restricting livestock mobility critical to sustainable livelihoods.

## Introduction

In Africa, livestock production contributes to income generation, and the livelihoods and food security of more than 70% of the rural poor^1^. More broadly, it is key to national development, for example through international trade. Endemic diseases, which are largely uncontrolled in these areas, not only affect global livestock economies^2^ but also threaten farmers’ subsistence^2,3^ through animal morbidity and mortality, and decreases in yield of animal products^3,4^.

The majority of the continent’s livestock populations are raised in traditional management systems. These husbandry systems rely exclusively on communal use of livestock resources (e.g. pasture and water)^5^. Agropastoral (comprising a combination of livestock rearing and cropping) and pastoral (relying almost exclusively on livestock’s contributions) systems are amongst the most predominant^5^. In East Africa, agropastoral systems account for 75% or more (e.g. > 80% in Tanzania) of livestock production, while 15% (14% in Tanzania) of livestock are raised in pastoral systems^5^. In both these systems, livelihoods depend on the ability to move livestock to communal areas to access resources critical for their survival, such as pasture and water^6^. Such movements tend to be local, but they cover variable distances depending on the season, as seasonality influences resource availability^7^.

Trade-related movements are motivated by financial gains associated with livestock transactions along market channels. Seasonality plays a role also in this type of movement. For example, demand for livestock and their products, hence movements to markets, may be driven by religious festivities^8,9^. Meanwhile, in humid and sub-humid zones, livestock are more often sold in the wet season due to the higher market value of fattened animals^8^, whereas, in arid and semi-arid zones, high mortality associated with frequent droughts in the dry season drives sales^8,9^.

Although these movements are essential to livestock survival and livelihoods, their role in the spread of infectious diseases is equally well recognised^10^. Trade-related movements facilitate spread over wide areas within short periods of time and are not limited by national borders^11,12^. For example, trade was considered epidemiologically important in within-country spread of peste des petits ruminants (PPR)^13^ and Rift Valley fever (RVF) in the Sahelian region of Mauritania and Senegal^14^. The dynamics of mobility in traditionally managed systems will likely affect disease spread differently. For example, sharing of resource areas has been mostly implicated in local spread^15^, but differing patterns of movement between agropastoral and pastoral communities^16^ may be associated with distinct risks. An understanding of local- and wide-scale movement patterns and drivers is important to facilitate the identification of key transmission nodes that should be monitored and targeted.

Many high-income countries maintain detailed digital datasets of animal movements between registered premises, often motivated by animal health concerns and enforced by regulation^17^. These data have allowed extensive use of network epidemiological methods to respond rapidly to animal disease outbreaks by modelling and tracing^18,19^. Beyond being consumers of network methods, the existence of these data has in many ways driven further method development, with network methods in veterinary epidemiology increasingly incorporating dynamic characteristics of the network and detailed structural features^20^. These methods have shown that networks of animal movements are key to understanding and controlling a variety of pathogens in livestock.

Gathering and maintaining comprehensive animal movement data in a low-income context so that network methods can be built is considerably more challenging. Furthermore, the unrestricted and unregulated nature of livestock movements in most of Africa compromises our ability to study their dynamics using methods commonly used in high-income countries. The lack of systematic livestock recording systems in Africa has been a major obstacle in studying movements and disease spread^21^. Previous research in East Africa focused mainly on understanding movements to markets using permits issued at trading points^22^. A major limitation of this approach is that such permits do not capture the origin of livestock, local movements (i.e. to and from communal areas) and informal transactions between communities^8,22^. Omitting these critical factors provides an incomplete picture of the actual scale and dynamics of movements. Local movements are particularly difficult to capture as they are not recorded officially and tend to depend on the availability of livestock resources such as pasture and water. As a result, they remain poorly understood. In particular, the extent of interactions at resource areas and the factors that influence them (e.g. seasonality and herd sizes) have neither been investigated in detail nor robustly quantified.

To overcome the difficulties in collecting data on local-level livestock movements, approaches that are reliant on consultation with local communities are critical, and they allow us to understand both the patterns of movements as well as their drivers. Participatory mapping methods, which involve consulting community stakeholders with relevant knowledge of the area^23^, are increasingly being used to identify risk areas for disease surveillance in limited-resource settings^24^. These techniques generate qualitative data, which have been typically used in a descriptive manner.

In this study we extend the use of participatory mapping data to reconstruct networks of livestock movements as reported by herders, encompassing areas of aggregation and mixing of animals (i.e. network nodes), and movement events in and out of such areas (i.e. network edges). We examine local and wide-scale (trade-related) movements in northern Tanzania, which are representative of the broader movement patterns in East Africa, to understand how seasonal availability of resources and trade influence the movements of rural livestock. Specifically, at the local level we focus on agropastoral and pastoral systems to (a) describe the patterns or herding characteristics and herd mixing at communal resource locations for rural livestock, (b) quantify village connectivity and identify key contact nodes that might represent important points of aggregation and (c) evaluate how factors such as seasonality, livestock numbers and corridors affect livestock networks. At a wide scale, in the agropastoral system, we examine the flow of traded livestock and estimate the probability and directionality of trade-related movements between rural and urban settings. Overall, our study identifies locations where livestock interact most, and trajectories and directionality of movements. Together, these suggest potential areas of disease transmission and highlight locations where targeted interventions tailored to specific livestock production systems may be focussed.

## Methods

### Local livestock movements

#### Locations of focus

This component of the study aimed at gathering and analysing data on local livestock movements in agropastoral and pastoral livestock production systems to understand herding characteristics and movements, reconstruct networks of village connectivity through key resource areas and evaluate factors influencing this connectivity. We focused on two districts of northern Tanzania to the west (Serengeti District, Mara Region) and east (Ngorongoro District, Arusha Region) of the Serengeti Ecosystem, which have dominant agropastoral and pastoral communities, respectively (Supplementary Fig. S1). Further detail on the study location is provided in the Supplementary Materials (Supplementary Methods).

#### Data collection

To gather data on local livestock movements, we employed participatory mapping approaches^24^, involving consultation with selected community stakeholders to identify resource areas and livestock trajectories on maps. The mapping exercises were conducted between July 2016 and June 2018. In Serengeti District, we targeted all villages (n = 97), while we focused on the Loliondo and Sale Divisions (46 villages) in Ngorongoro District. A village was considered a mapping unit because it is the smallest administrative unit, and this enabled us to capture the finest level of movements within the entire village. In addition, livestock from neighbouring households are often grouped during herding, which create a homogenous mix within the village. In each village, participatory mapping sessions involved 8–10 participants, comprising local livestock field officers, village chairmen, sub-village heads, prominent livestock traders, livestock movers and livestock keepers. These stakeholders were selected based on their knowledge of livestock matters, including location and use of communal areas such as grazing lands and water holes, which they were asked to draw on maps (Supplementary Figs. S2–S4) (see Supplementary Methods for full details of the participatory mapping process). During these sessions, qualitative data were also collected on: (a) preferences for grazing areas and reasons for these; (b) village-level herd sizes and the maximum number of livestock herds that could be moved as a single unit to resource areas; (c) predominant agricultural practices; (d) settlement types; (e) source of livelihoods (i.e. livestock or crops) and herding patterns (e.g. availability of land-use plans, seasonal resettlements); and (f) season classifications and their corresponding months.

#### Analyses of local livestock movement data

##### Data processing

Metadata containing details on how each village move their livestock to communal areas were extracted manually from digital field notebooks, voice recorders and hand-drawn maps and entered in text format in Excel spreadsheets. The community-drawn paper-based maps showing location of key resource areas were georeferenced by initially uploading scanned versions of the maps into QGIS via ‘Georeferenced GDAL’ (a core plugin)^25^ and then manually entering coordinates on the digitised maps to complete the georeferencing process. Each georeferenced tiff (GeoTIFF) file was validated by a two-way process: (1) overlaying the points that were collected on the ground using a hand-held Global Positioning System (GPS) device during the field observations, and (2) overlaying on Google Earth satellite images to confirm the absolute position of the hand-drawn shapes of resource areas.

After validation, GIS shapefiles (comprising polygons, lines, and points) were created for each type of resource area (e.g. pasture or water) and livestock routes. The resource areas (pasture, water, dips and salt points) were further organised into three tables containing labels of key features, which were linked into an SQL database using the tidyverse package version 1.3.0^26^, in the R programming language version 3.6.1^27^ for data cleaning and analyses. For each village, the three tables contained: (a) livestock movement events for each resource area, including frequency of movements by season/month (e.g. daily movements or camping), the number of livestock moved to the area, and other villages’ livestock that used the area; (b) detailed information on each livestock resource area, including name, type, size, relative position (i.e. x/y coordinates), villages where they were located or to which they were closest, and metadata to allow cross-referencing with the other tables in the database; and (c) digitised information for each location from QGIS, including centre waypoints and shapefiles for each resource area.

##### Herding characteristics and patterns of movements

Qualitative data such as reasons for preference of certain pasture or water locations, patterns of livestock herding (e.g. mixed or non-mixed small and large ruminant herding), duration of herding and relocation by pastoral migrating herds were analysed to establish patterns of livestock keeping and herding characteristics that might affect movement, and to gain insight that would enable us to correctly interpret the result of the network analyses. We include these interpretations in the discussion. Full details of the qualitative data extraction and analyses performed are included in Supplementary Methods. In brief, qualitative data related to movement patterns and characteristics of livestock herding were coded using simple key words/phrases derived from the participatory mapping sessions^28^. For example, livestock grazing around rivers was coded as “river-bank grazing” and relocation of herds to neighbouring villages for several weeks as “camping”. These descriptive key words helped establish patterns and build themes that were consistently applied across villages (see Supplementary Table S1). Summary analyses were performed by grouping the key themes to identify movement types by season/month, and to establish their patterns and frequency, and reasons for movements and for preferring certain areas.

##### Village connectivity and central nodes of aggregation

We reconstructed two types of networks to demonstrate the sharing of resource areas between villages and from where networks measures were calculated. The first type, used to illustrate village connectivity (i.e. the strength of contacts or connections in the network), was directed and bipartite, that is, there were two types of nodes, and all edges were between a node of one type and a node of the other type. Nodes were resource areas and villages, and edges represented connectivity between a village and a resource area if that village used that resource area. The multiplicity of each edge (strength of connection) represented the frequency of use of the resource by the village. The second type of network was an undirected weighted network in which the nodes were villages, and the edge weights represented the number of shared resources between two villages and the frequency of their use—an indicator of the strength of contact between villages (i.e. village connectivity). This type of network was used to identify important nodes, based on centrality measures^29^, and to understand differences across management systems and seasons. Briefly, centrality measure degree indicates the number of connections to or from a node (village) in a defined period (e.g. day, month or year), betweenness describes the frequency by which a node falls between pairs of other nodes on the shortest path that connects them and eigenvector values indirectly measure centrality determined by the centrality scores of the nodes to which the node of interest is connected to. The network measures were weighted and normalised^30^ (see Supplementary Methods and Supplementary ﻿Table S2 for full details of the measures and all terms used in the study). Graph visualisations and analyses were performed in the R Programming Language using the i-graph package version 1.2.4.1^31^.

##### Factors affecting networks of local livestock movements

To investigate factors affecting the properties of local movement networks, the density (number of edges in the observed network relative to the total number of possible edges in a completely connected network), and spatial characteristics such as number and distance of connected villages, district’s livestock population numbers and seasons were calculated and compared across time and livestock production systems. First, we regenerated the networks for different seasons and systems, and calculated a suite of network measures for pastoral and agropastoral systems. Second, we calculated distributions of geographic distances of local movements over systems and seasons. Finally, we used the Spearman correlation test to assess the influence of village livestock populations on the local movement network measures. For example, we broadly assessed if village network properties were associated with the number of livestock. All analyses were performed in the R Programming Language^27^.

### Wide-scale (trade-related) livestock movements

For this component of the study, we analysed livestock movement permits to understand the dynamics of trade-related movements and the flow of movements connecting rural and urban settings.

#### Locations of focus

We focused on four largely agropastoral districts in the Mara region, Serengeti, Bunda, Butiama and Tarime, comprising both urban and rural wards (Supplementary Fig. S1). These districts are the only human-populated areas with higher livestock density (i.e. number of livestock per km^2^) and thus higher traffic adjacent to Serengeti District, when compared to the other districts^32^, and were therefore selected to investigate inter-district connectivity through livestock trade movements.

#### Data sources

Livestock movement permits (LMPs), issued by the government at trading points in major livestock markets for the collection of levies or tax on traded livestock, were gathered for the period January to December 2017. Permission was granted by the Tanzanian Ministry of Livestock and Fisheries through the Zonal Veterinary Centre in Mwanza. Full detail of methods and information extracted from the permits is provided in Supplementary Materials (Supplementary Methods).

#### Analyses of trade-related livestock data

Analyses were performed to quantify major livestock flows, from origin to destination, between districts in the Mara region and the extent to which traded livestock were moved to districts in other regions within and beyond Tanzania.

##### Network of traded livestock

To evaluate the strength of contacts and identify central villages, a directed weighted network was constructed for each district. Within the network, nodes were villages of livestock origin or destination, or market locations, while the edge weights were a function of the number of livestock traded between villages. Note that, before trade, villages where markets were located were classified as destinations, but these became villages of origin after livestock had been traded. The origin and destination of traded cattle were represented in a circular map. We calculated relevant centrality measures for each node (Supplementary Table S2) with the aim of determining their importance in the network^30^ and potential targets for disease-control interventions. Correlations between network metrics were examined using Spearman’s correlation test statistic. All network visualisation and analyses were performed in the R Programming Language using the i-graph package, version 1.2.4.1^31^.

##### Probability and directionality of movements

A gravity model was used to estimate between-village flow of traded livestock in the Mara region. This type of model was primarily developed in the socio-economic field to measure mobility of people and its economic relevance^33^. Here, the model assumes that the flow of a commodity from a point of origin to an area of destination is influenced by several factors including the population at the origin and destination, and the distance. The gravity equation was linearised by a log-transformation, and the coefficients were estimated using a generalised linear model as described by Nicolas et al*.*^9^ The model investigated movement events between villages using two approaches: the first estimated the probability of livestock moving from village “i” (origin) to “j” (destination), while the second determined the factors that affect the volume of traded livestock or traffic from village “i” (origin) to “j” (destination).

For the first approach, all possible pairwise combinations of villages or towns in the study district were identified, and a fitted binomial generalised linear mixed model (GLMM) with logit link function was used to estimate the coefficients. The binomially distributed response variable included: all pairs of villages (nodes) that had at least one movement event between them (coded as 1); and all other pairs of villages without any movement events (coded as 0). The second approach allowed us to further differentiate the factors influencing the trade probability from those influencing its volume. Hence, a zero-truncated negative binomial (ZTNB) GLMM with the number of traded cattle as response variable was used to estimate the volume of livestock trade between the two locations (village/town of origin and village/town of destination). For all models, in both approaches, eleven combinations of predictors (Supplementary Table S3) were considered for both origin and destination villages. Variables were dropped in a stepwise process with the least significant variable (*p* > 0.05), as determined by likelihood ratio testing (LRT), being dropped first. To improve model convergence, continuous explanatory variables were scaled (to have a mean = 0 and a standard deviation = 1) and the Bound Optimisation by Quadratic Approximation (*bobyqa)* model optimiser was used^34^.

### Ethics statement

The study, including protocols for the focus group discussions, was approved by Tanzanian Commission for Science and Technology, which is the national institutional review board, with reference numbers: 2016-93-NA-2016-87 and 2017-284-NA-2016-87. The approval also allowed and enabled relevant local and district authorities to support the research components undertaken in their communities, and the study was performed in accordance with relevant guidelines and regulations. All focus group participants were informed about the study through a Participant Information Sheet developed for this purpose. Participants were all adults and participation was voluntary. Written and/or verbal informed consent was obtained before proceeding. Permission to extract data from the livestock movement permits was obtained from the Tanzanian Ministry of Livestock and Fisheries (Permit No: PA 15/116/02/23).

## Results

### Local livestock movements

#### Herding characteristics and patterns of movements

Compared to agropastoralists, pastoral households kept greater (by 50%) numbers of livestock, particularly small ruminants (75% more). Livestock sales were the main (up to 100%) source of livelihood for pastoralists, while agropastoralists derived more than 50% of their income from crops compared to livestock (Table 1). Agropastoralists typically engaged in daily movements to resource areas, while pastoralists established temporary settlements based on availability of livestock resources (Supplementary Results and Supplementary Table S4).

**Table 1:** Summary of livestock populations in the study area based on local government-level data, and agropastoral and pastoral livestock and crop keeping characteristics based on participatory mapping discussions.

Category	Agropastoral (Serengeti)	Pastoral (Ngorongoro)
Total livestock kept by district		
Cattle	400,823	379,988
Goats	148,294	529,012
Sheep	194,855	531,191
Village-level livestock population estimates		
Cattle		
Mean	4224	8261
Median	3737 [IQR: 2242–5159]	7248 [IQR: 3431–11,393]
Goats		
Mean	1564	11,500
Median	1315 [IQR: 832–1858]	7585 [IQR: 4465–13,258]
Sheep		
Mean	2052	11,548
Median	1504 [IQR: 965–2192]	6944 [IQR: 4139–11,023]
Mean household-level herd size		
Cattle	50	250
Goats	20	350
Sheep	10	200
Main source of livelihood		
Livestock	Less than 50%	75–100%
Crops	More than 50%	0–25%

IQR is the interquartile range of the median values for the livestock populations. Note: mean household-level herd size was based on the community perception.

#### Village connectivity and central nodes of aggregation

Descriptive network analyses performed on data from each study village (n = 143) revealed that connectivity (that relates to the edge weight or connection strength and is calculated as the proportion of edges connected to a node relative the full network) (Supplementary Figs. S5 and S6) was either 40% direct , i.e. at resource areas shared between villages (Supplementary Fig. S6), or 60% indirect, i.e. when mixing of herds between two villages was mediated by a third village (Supplementary Fig. S6). The higher percentage of indirect connections indicates that communal livestock resources are located in fewer villages. For example, some villages go to another village to access both pasture and water (Supplementary Fig. S6B).

Overall, four types of resources (i.e. grazing areas, watering locations, salting, and dipping points) drove connectivity disproportionally between villages through livestock movements in both agropastoral and pastoral systems, which suggest that different levels of risk would be associated with each resource areas. In agropastoral systems, movements to grazing areas accounted for almost half of all between-village connectivity (49%), followed by water points (25%), dipping points (14%) and salt points (12%). In pastoral systems, movement to watering locations accounted for the greatest connectivity (43%), followed by grazing (35%), salt points (21.8%) and dipping points (0.2%) (Supplementary Table S5). For all networks, only a few villages controlled the majority of the connections at resource areas (Supplementary Figs. S7–S9), with network densities being higher in pastoral compared to agropastoral systems (Supplementary Table S5). Full details of the four types of resource areas and associated contact networks are described in Supplementary Results (Supplementary Box S1).

#### Factors affecting networks of local livestock movements

Between-village connectivity through key resource areas and location of central nodes (i.e. “important” villages that control the movement of livestock) in the network of local movements showed heterogeneities across production systems, seasons and livestock numbers. In this section, we investigate these variations and factors driving them in more detail.

##### Effects of livestock production systems

Despite the fact that there were twice as many villages (nodes) in agropastoral compared to pastoral settings, i.e. 97 versus 46, there were fewer than half as many edges (village-to-village connected links through shared resource areas), i.e. 11,906 versus 23,128 (Supplementary Table S6), which resulted in a denser and more connected network in pastoral systems (Supplementary Tables S5 and S6, and Supplementary Fig. S7). The unique contacts (unweighted mean degree values) showed (broadly) that villages in the pastoral community have more links than agropastoral villages (Supplementary Tables S5 and S6). Centrality measures showed variations across seasons for each network, with a small number of villages exhibiting relatively high values (Supplementary Table S6 and Supplementary Figs. S7 and S10), reflecting their influence over others and important reference points in the networks.

##### Effects of seasons

**Figure 1:**
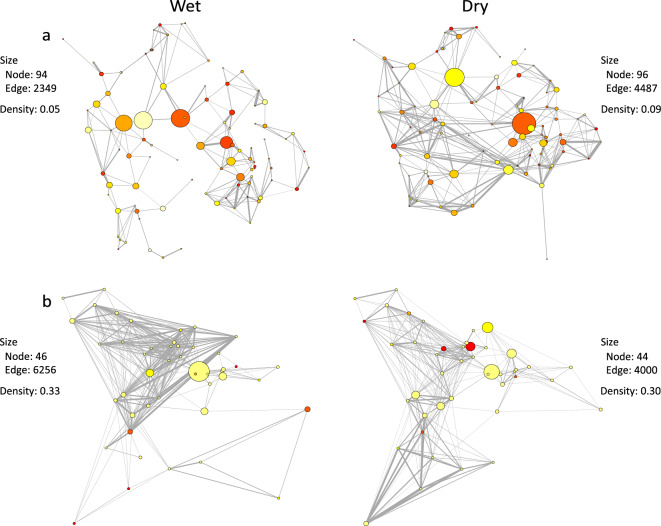
Spatial livestock movement networks of village connectivity showing variation in contact patterns across seasons and production systems [(**a**) agropastoral and (**b**) pastoral)] investigated in northern Tanzania. The nodes (circles) are villages in their geographical position. The grey edges represent shared resource areas among connected villages (degree). The node size is proportional to the network centrality measure betweenness for the village and colours relate to the centrality measure eigenvector with red being the highest value (see Supplementary Materials, Table S2 for detailed definitions of centrality measures).

The extent of between-village connectivity, including mean pairwise geographic distance, varied across seasons in both agropastoral and pastoral systems, although these variations differed between the two systems. In both agropastoral and pastoral systems, the relative influence of villages as shown by their centrality values varied by season: villages that were central (i.e. had high betweenness and eigenvector centrality values) in the wet season differed from central villages in the dry season (Fig. 1).

In agropastoral settings, there were fewer connections and inter-village livestock interactions (weighted degree) in the wet compared to the dry season with herd aggregations mostly observed within rather than between villages (Fig. 1a). As a result, some villages and resource areas were not connected in the wet season (Supplementary Fig. S5) and the associated network was less dense at this time of the year (Supplementary Table S6).

The reverse was the case for pastoral settings where there was a greater number of village contacts in the wet than in the dry season, resulting in an overall denser network in the wet season (Fig. 1b, Supplementary Table S6). However, during an exceptionally dry period associated with a drought the network showed high connectedness and density (40%).

Movements involving agropastoral villages showed higher geographical proximity, while pastoral livestock herds were moved to more distant areas. For example, the distance between connected villages was almost three times higher in pastoral (median: 14 km, IQR: 10–25 km) compared to agropastoral (median: 5.6 km, IQR: 2.5–7 km) systems (Supplementary Fig. S11).

**Figure 2:**
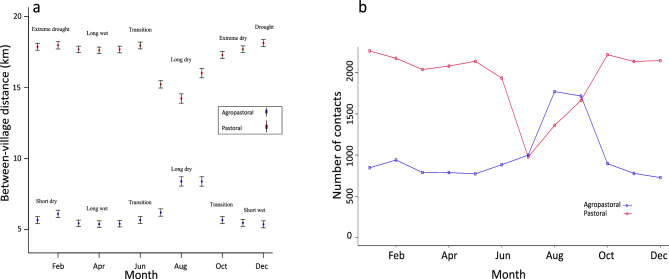
Distance and number of connections between villages that share resource areas by month and across livestock management systems. (**a**) Between-village distance across seasons, including a period of drought. The vertical bars are confidence intervals of the mean. (**b**) Changes in the number of contacts between villages every month across production systems. In the study sites, the period March—May typically corresponds to the long wet season, while from July to September the long dry season occurs. The months of November and December generally typify the short wet season, which in this study failed in the pastoral setting and was replaced by a drought period that extended into January–February, while the agropastoral setting was typified by the short dry season that typically occurs at this time.

Seasonality influenced the distance travelled between villages, but variations were observed between agropastoral and pastoral systems (Fig. 2). Livestock herds travelled significantly less in the wet (mean pairwise distance of connected villages ranging from 2.1 to 5.8 km) compared to the dry (9.0 km) season in agropastoral settings (Fig. 2a). In pastoral settings, study participants reported a more complex livestock movement scenario because they typically use temporary settlements and camping areas across rangelands both in the wet and dry seasons (Supplementary Table S4). However, a clear pattern was observed with significantly longer mean pairwise distances (up to 18.5 km) among connected villages in the wet compared to the long dry season (14.4 km), except during a drought when herds were taken further distances (up to 19 km) (Fig. 2a). The number of contacts had a similar seasonal pattern with the distance between connected villages in both agropastoral and pastoral systems. For example, villages in the pastoral systems had twice more contacts in the wet compared to the dry season (Fig. 2b).

##### Effects of livestock population numbers

The eigenvector centrality value of pastoral livestock movement network was the only measure associated with the number of livestock (i.e. sum of cattle, goats and sheep) in the village. This positive correlation (R = 0.45, *p* < 0.05) suggests that in the pastoral settings, a village eigenvector value increases with livestock population (Supplementary Fig. S12).

### Wide-scale livestock movements

Overall, 67,110 livestock (cattle: 45,947; goats: 15,882; sheep: 5281) were traded across 38 markets, with the highest numbers in the Serengeti District and lowest in Bunda Town Council (Supplementary Table S7). The total volume of cattle traded was twice more than small ruminants combined, although amongst the latter more goats were traded compared to sheep. All district markets had a small proportion of livestock that originated from neighbouring districts, except for Tarime where most traded cattle came from other districts (Supplementary Table S8). Median distances from villages of primary origin to markets of destination ranged from 9.7 to 22.0 km, depending on the district.

#### Network of traded livestock

**Figure 3:**
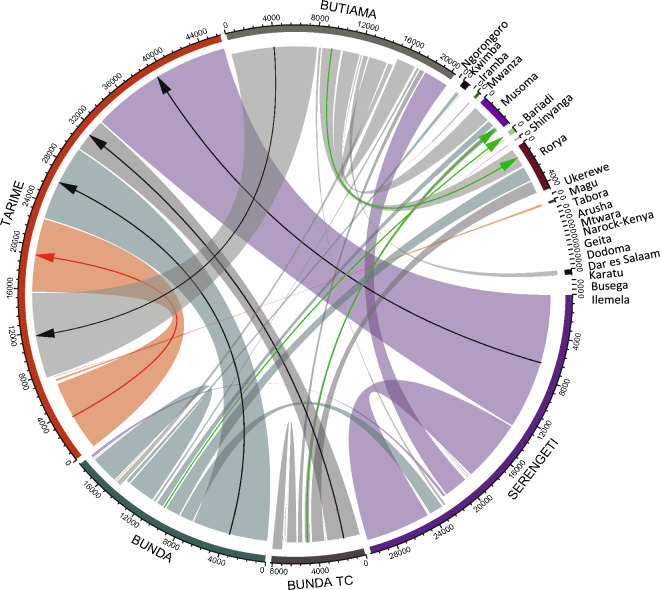
Flow and traffic of traded livestock in the Mara region, northern Tanzania. The five districts where livestock markets were located and traded from are indicated in uppercase. The axis has colours (which is unique for each district or town where cattle were either traded in or moved to), and a scale (which starts from 0 and with each unit representing 800 livestock). The scale on the axis indicates the number of livestock moved. Outgoing livestock flow from each trading point (origin) starts from the higher point on the axis or base of the district where markets were located, while the in-flow into the district or town (destination) is closer to the axis. For example, the axis for Serengeti district shows that 30,400 livestock were transacted, approximately 22,400 were out flow with the majority to Tarime district, and 8000 in flow. Likewise, the axis of Tarime district shows that 46,000 livestock were transacted, approximately 7200 were traded in Tarime and remained in the district, and the rest were inflow from other districts. The black coloured arrows indicate major movements from other districts to Tarime, while the red coloured arrow indicates that almost all of the traded livestock in Tarime were received or remained in the district. The green coloured arrow shows other major towns or cities where livestock were moved within the Mara and neighbouring regions.

Each district had several traded livestock moved to multiple destinations. For instance, 60% of traded livestock were moved to Tarime town, 20% to slaughterhouses located in towns in the Mara region, 10% to various districts in other regions (including 1% that was moved across the border to Narok (Kenya), and the remaining 10% were re-introduced to villages within the Mara region (Fig. 3).

The heterogeneous (i.e. variations in volume of livestock between origin and destination locations) structure of the network of traded livestock showed that all districts in the Mara region were interconnected, with various trading links to neighbouring regions including those across the border (e.g. Narok in Kenya).

**Figure 4:**
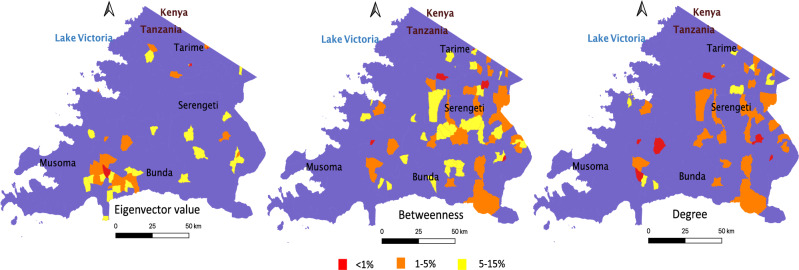
Spatial location of villages with the highest centrality measures based on a full directed network of origin and destination of traded livestock in the Mara region of northern Tanzania. All measures were weighted and normalised (see Supplementary Materials, Table S2 for detailed definitions of centrality measures). The shapefiles were based on 2012 Tanzania census, obtained from Tanzania National Bureau of Statistic. The map was developed in Quantum geographical information system (QGIS), version 3.18^25^.

Village-level variations were observed in centrality values across network metrics, with a few villages having consistently high centrality values across measures (Fig. 4).

Villages in the top 5% weighted degree and betweenness values had markets, and village degree and betweenness values were positively correlated (R = 0.49, *p* = 0.007) (Fig. 4). In addition, for betweenness, the centrality values were positively correlated with market size (R = 0.40, *p* = 0.009), while the eigenvector centrality values were positively correlated (R = 0.29, *p* = 0.001) with village in-degree (i.e. the number of livestock that were moved into the village).

#### Probability and directionality of movements of traded livestock

The 67,110 livestock traded involved 1770 unique movement events between locations of origin and destination. Three (pairwise distance between origin and destination, market presence at origin, and market presence at destination) out of the eleven fitted explanatory variables (Supplementary Table S3) significantly affected the probability of a movement of traded livestock between villages (Supplementary Table S9), while five fixed effects estimated the volume and direction of livestock movements between locations (Table 2).

**Table 2:** Significant explanatory variables from the final Zero-truncated Negative Binomial regression (ZTNB) model that estimated the volume of traded livestock movements between location (village/town) pairs.

Variable	Estimate (95% CI)	Ratio (95% CI)	*p* value
Pairwise distance between origin and destination	−0.95 (− 1.13 to 0.77)	0.39 (0.32–0.46)	< 10^ − 9
Livestock population size at destination	0.24 (0.15, 0.32)	1.27 (1.16–1.38)	< 10^ − 6
Market presence at destination (Yes vs No)	1.06 (0.92, 1.21)	2.89 (2.51–3.35)	< 10^ − 9
Urban destination (Yes vs No)	0.67 (0.60, 0.74)	1.95 (1.82–2.10)	< 10^ − 9
Human population at destination	0.54 (0.32, 0.76)	1.71 (1.37–2.14)	< 10^ − 4
Pairwise distance: urban destination compared to rural	− 0.17 (− 0.23 to 0.11)	0.84 (0.79–0.89)	< 10^ − 5

All continuous variables were scaled to mean zero and one standard deviation. CI denotes Confidence Interval.

The probability of livestock movements from an origin to destination and the volume of livestock that was moved reduced significantly as the distance between them increased (Table 2 and Supplementary Table S9). The estimated ratio indicated that the volume of movement decreased by a factor of 0.39 (CI 0.32–0.46) for every standard deviation increase in distance between villages. At the same time, the volume of traded livestock was 1.95 (CI 1.82–2.10) times higher if the destination was an urban compared to a rural location, and 2.89 times higher for a destination with markets. However, the significant interaction term indicated that the volume of livestock would reduce by a factor of 0.84 per one standard deviation of increasing distance to the urban destination (Table 2).

## Discussion

Using network modelling on participatory mapping data combined with trade information, we investigated livestock movements in two livestock production systems in rural Africa, agropastoral and pastoral, in order to identify locations of greatest traffic, and hence of high disease transmission potential. Patterns of local movements and village connectivity, which were driven by the need to access key livestock resources, varied across production systems and seasons. Agropastoral villages were geographically closer with fewer inter-village contacts and herds travelled less in the wet compared to the dry season. This pattern was reversed in pastoral areas, with the exception of extremely dry weather during which movements were greater and villages were highly connected. Village “centrality” (i.e. importance) in livestock networks also varied across production systems and seasons. In addition, in pastoral settings, livestock numbers influenced connectivity, whereby villages with large livestock populations were more connected to each other, likely because pastoralists tend to keep larger herds compared to agropastoralists. Trade was another important reason for livestock mobility: it connected all northern Tanzanian districts examined, including across the border to Kenya. The presence of markets and reduced distance between origin and destination increased the probability of movements both in villages and urban centres. However, the volume of traded livestock was higher if the destination was urban, with rural areas acting as movement “sources” and urban destination as “sinks”—only 10% of animals returned to their rural origin. Our results indicate that interventions targeted at the correct time (i.e. prior to and after the wet season in pastoral and agropastoral areas, respectively) and at focal locations (i.e. locations of high centrality values) may be a cost-effective approach to limit infection without restricting livestock mobility critical to sustainable livelihoods.

Access to livestock resources such as grazing, water, dipping points and mineral salt deposits is essential for livestock survival and a major driver of local movement. In our study, the movements to pasture and water points tended to occur daily, with longer distances and higher intensity of encounters in pastoral compared to agropastoral systems. Watering resources were generally of greater importance to pastoralists because dehydration is considered to have a greater risk of fatality than starvation. Indeed, pastoralists keep more small ruminants than cattle (Table 1) as they are more resilient to harsh weather conditions^35^. Generally, agropastoral herds spend more time at dipping and grazing locations compared to watering points, likely because only a few dips are functional and this creates long queues at the remaining dips^7^. Each week, up to 13 out of 93 villages in the agropastoral system were connected at a single dipping point, resulting in high livestock traffic at these locations. In contrast, access to dipping is logistically more challenging for pastoral communities, especially during periods away from permanent settlements. Pastoralists’ decisions on movements to grazing areas are typically influenced by availability of mineral salt deposits, even if vegetation levels are low, whereas agropastoralists make less use of this resource. Although grazing and watering locations might pose high risks of livestock disease spread due to daily mixing in both production systems, in agropastoral systems the congregation of large numbers of livestock herds at dipping points is of equal concern.

Across sub-Saharan Africa, the frequency of use of resource areas is linked to resource availability, which in turn is influenced by season^36^. Although two distinct seasons, wet and dry^37^, are generally recognised, rainfall patterns are becoming increasingly inconsistent and unpredictable, resulting in long spells without rain in the wet season, and extended dry seasons and droughts. In our study, changing patterns in rainfall drove unplanned livestock movements, especially during the drought experienced by the pastoral communities (Fig. 2), which could increase risks of disease spread. Seasonal variations might influence production systems, hence disease spread, differently. For example, higher movements and contacts between villages in the wet compared to dry season in pastoral systems (Fig. 2) might explain the observed higher prevalence of foot-and-mouth disease (FMD) in the wet compared to the dry season in these systems^38^.

In the agropastoral setting, village encounters had a strong geographic dimension across all seasons with relatively short distances between connected villages (Figs. 1 and 2). Livestock return daily from communal resource areas because of the proximity of human settlements to these areas (Supplementary Table S4). Therefore, livestock herds from villages located within 6.0 km have a high probability of encountering each other at communal resource areas. In dry conditions, between-village distances were wider (15.0–41.0 km) because of the need to move animals to more distant resource areas, including those located in other villages. Similarly, at the village level, networks of herd encounters at communal areas in the dry compared to the wet season were more complex (i.e. in terms of number of nodes and edges), resulting in more inter-village contacts and greater density (Supplementary Table S5). This pattern is consistent with other studies^7,39^: as pasture and water resources become scarcer, an increasing number of herds are forced to share the few remaining resources and to travel longer distances to reach them. In contrast, the abundance of pasture and water in the wet season means that most villages are self-sustained. Therefore, in the wet season, when water is widely accessible, agropastoral livestock travel shorter distances with fewer and less frequent interactions mainly within villages.

Pastoralists in the study system are primarily livestock keepers with no crop farming. However, this concept of pastoralism is changing as observed in our study whereby pastoral nomads that have no permanent settlements are few (Supplementary Table S4), and an increasing number of pastoral communities are adapting their farming systems to accommodate socioeconomic, political and environmental challenges by including some cropping. As mentioned in the results (Table 1), pastoral communities now cultivate crops, which in some cases contribute up to 25% of their household income. Unlike the patterns observed in agropastoral systems, migrating herds in pastoral areas were involved in complex multi-village connectivity, which resulted in long-distance connections with no specific geographic dimensions, and higher network density. A village in these areas is usually connected to multiple (mean: 20) other villages due to the continuous re-settlement of herds to temporary locations as pasture and water become depleted, especially during the dry season. Livestock herders travel long distances looking for suitable grazing while camping en route, resulting in an average distance of 17.0 km among connected villages (Fig. 2). If the short rains fail, as in our study, herds are moved even longer distances (between 40 and 100 km), often to multiple areas and for prolonged periods depending on the extent of the drought. At temporary locations, where herders may settle for several weeks until forage or water levels become low (Supplementary Table S4), herds are likely to interact with others from multiple villages, including distant ones, that are migrating for similar reasons. Such complex inter-village contacts involving several links explain the high-density network obtained in the pastoral settings. Movements to temporary sites comprising multiple villages are common at the beginning of both wet and dry seasons, although valuable stock (e.g. young calves and milking cows) are usually left at permanent settlements. The long distance between connected villages in the wet-to-dry transition period (June) coincides with the return journey back to permanent settlements. By July, following the long-wet season, crop residues are sometimes available to livestock, but only in areas where pastoralists engage in crop-related agriculture. During this time, herds are moved and returned daily as reflected in the reduced inter-village distance and number of contacts (Fig. 2).

A further explanation for the differing patterns observed in the pastoral settings relates to local disease avoidance strategies specific to malignant catarrhal fever (MCF). MCF virus, which causes a fatal disease in livestock, is released by migratory Serengeti wildebeest *(Connochaetes taurinus mearnsi)* during the calving period in the wet season^40^. During this period livestock and wildebeest share the large expansive grassland pasture of the Serengeti plains. To reduce livestock contact with the virus, pastoralists normally relocate herds multiple times to avoid calving wildebeest. This may further account for the long distances and high number of village contacts observed in the wet compared to the dry season in pastoral settings. The differences in the contact structure of livestock movement networks across seasons and production systems provided some understanding of how endemic diseases might spread in these systems. For example, higher density networks comprising multiple villages and longer between-village distances would suggest that livestock diseases might spread faster between communities in pastoral compared to agropastoral systems.

The volume and directionality of livestock trade-related movements were driven by presence of markets, distance between origin and destination, and type (rural or urban) of origin and destination (Table 2). Given fluctuations in market prices, farmers may prefer to sell in their local markets, as this removes the need to travel long distances to distant markets that may not guarantee better prices. The majority (90%) of traded livestock in this study were destined to markets, particularly urban, although this proportion also included some animals moved to slaughterhouses located in towns or cities. Overall, nearly all traded livestock originated and remained within the study region (Mara) (Fig. 3), with only 10% being moved to other regions. A small proportion (10%) of animals likely returned to individual households in villages and were restock into local herds, or were sold informally to other livestock traders in the rural communities. It is also possible that they might have been used for local events such as marriage ceremonies, for example as dowry.

Consistent with the direction of travel, the presence of markets drove the importance of locations (i.e. centrality) in networks of traded livestock. Specifically, locations with markets, or without markets but that received a large volume of livestock from large markets (e.g. slaughterhouses and livestock holding grounds), were at the top 5% of the centrality measures. Urban locations with high human populations but low livestock had high measures of centrality, likely because of the demand for livestock products. These findings are in line with previous studies, where the destinations of long-range livestock movement were associated either with markets or with locations connecting markets (e.g. farms or holding grounds). Similarly to our study, these locations in other studies displayed high betweenness centrality values^29^ and high eigenvector index values^11^, and are ideal locations where intervention should be focused^41^.

Seasonality can drastically change eigenvector centrality values due to dynamics of trading patterns^42^. In our study, more livestock were sold in the wet compared to the dry season, but we did not detect any consistent seasonal pattern. A previous study in central Africa also reported higher livestock sales in the wet compared to the dry season^8^. However, a study in Mauritania reported higher sales in the dry season^9^, due to a major festive event. This suggests that sociocultural factors that coincide with specific seasons may be more likely to influence the timing and type of livestock trade in any given area, rather than season alone.

## Conclusion

Our connectivity networks incorporating local and wide-scale livestock movement data enabled us to identify central nodes and times that might be epidemiologically important. Specifically, we have identified (a) locations both in rural and urban areas that drive livestock movements, (b) times and seasons when livestock movement is busiest, and (c) a rural-to-urban trajectory of trading driven by regional demand.

The findings enable a targeted approach to restricting livestock movement or other relevant interventions, such as strategic vaccination at key locations or times, or market closures. Fragmentation of the network by “removing” central nodes would decrease the probability of large epidemics^8,43^. For example, studies conducted on cattle trading networks in Africa have shown that tackling 20% of nodes (villages) with high betweenness values or degrees will reduce epidemic size by 80% when compared to randomly targeted nodes^8^. Other studies elsewhere reported that targeting 10% of farms with high betweenness values or degrees will reduce endemic disease prevalence by 80% when compared to randomly targeted nodes^44^. Villages with hub characteristics (villages with high centrality measures) differed across seasons (Fig. 1) because their main properties (e.g. presence or absence of livestock resources) are also affected by seasonal changes, and few villages had high centrality values across all seasons.

The timing of such interventions would need to be tailored to the differing patterns we observed (specifically, prior to and after the wet season in pastoral and agropastoral areas, respectively). For diseases that spread fast, such as FMD, control measures would have to be season-specific and target villages that are important in the epidemic window. For a slow spreading disease, such as bovine tuberculosis, that might require a long-term control plan, interventions that focus on important areas regardless of the season might be more appropriate. However, prophylactic interventions such as routine vaccinations might lead to better outcomes if focused on areas that are important all year round. Wet-to-dry transition periods likely present the highest risk of spread for pastoralists because herds from villages connected during the wet season return to permanent settlements.

## Supplementary Information

Supplementary Information.

## Data Availability

We do not have Tanzanian government permission to publicly share trade data extracted from livestock movement permits. All other data and codes used in this manuscript are available as supplementary materials.
