# Supplementary material for: Local and wide-scale livestock movement networks inform disease control strategies in East Africa
Source: Sci Rep. 2023 Jun 14;13:9666. doi: 10.1038/s41598-023-35968-x (PMC10267211; doi:10.1038/s41598-023-35968-x)
Supplement: Supplementary file 1 — Supplementary Information. [file 41598_2023_35968_MOESM1_ESM.pdf]

# Local and wide-scale livestock movement networks inform disease control strategies in East Africa

Divine Ekwem[1,3*], Jessica Enright[2], J. Grant C. Hopcraft[1], Joram Buza[3], Gabriel Shirima[3], Mike Shand[4], James K. Mwajombe[5], Bernard Bett[6], Richard Reeve[1**], Tiziana Lembo[1**]

*Corresponding author

**These authors contributed equally

[1]Boyd Orr Centre for Population and Ecosystem Health, School of Biodiversity, One Health & Veterinary Medicine, College of Medical, Veterinary & Life Sciences, University of Glasgow, Glasgow, United Kingdom

[2]School of Computing Science, University of Glasgow, United Kingdom

[3]Nelson Mandela African Institution of Science and Technology, Arusha, Tanzania

[4] School of Geographical & Earth Sciences, University of Glasgow, United Kingdom

[5]Tanzania Agricultural Research Institute, Ministry of Agriculture, United Republic of Tanzania

[6] International Livestock Research Institute, Nairobi, Kenya

## Supplementary Materials

## Supplementary Methods

### Local livestock movements

**Participatory mapping**. *Location of focus.* The Serengeti District has a human population of 250,000 [1] and a livestock density of 84/km$^2$ (Unpublished National Livestock Branding Report) within an area of 9,000 km$^2$. Ngorongoro District is inhabited by 175,000 pastoral people in an area of 14,036 km$^2$ [1], with livestock density of 103/km$^2$ (Unpublished National Livestock Branding Report).

*Mapping tools.* Quantum geographical information system (QGIS), version 3.18 [2] software was used to construct maps at a variety of scales (1:2,500, 1:5,000 and 1:10,000) incorporating downloaded Google Earth satellite imagery on a gridded map base The image dimension was 1 metre by 1 metre at each scale (representing 2.5 km, 5 km and 10 km on a side, respectively), which ensured ease of sharing, and visibility and recognition of topographical features such as human settlements, hills, rivers/dams, roads, crop lands, utility centres etc (Fig. S2). In addition to these maps, wider scale (1:50,000) published topographic maps (with the addition of Tanzanian 2012 Census village boundaries) were printed at 1 metre by 1 metre (50 km on a side) and used to visualise livestock mobility corridors spanning across several villages, village borders such as hills and contours, large rivers and springs, rangelands, grass plains etc. (Fig. S3). All maps used were digital and their built-in coordinate systems were converted to Universal Transverse Mercator (UTM) projection and WGS84 datum to allow the participants to consistently identify the location and distances of key features across maps, aiding the mapping process. This provided a means to locate and estimate the size of livestock resources and crop lands more precisely.

*Data collection.* Before visiting each village, baseline information about grazing and watering patterns and other livestock-keeping characteristics was gathered for each village through consulting with the District Veterinary Office.

At village-level, a quick exploration of the digital maps was used to orientate participants and for map validation. To allow them to freely express themselves, the local language (Swahili and Maasai) was used for the discussion. Participants agreed on a clear layout of the community by combining the Google Earth images and topographic maps (Figs. S2 & S3). Subsequently, all relevant features and key resources were identified and located on the printed maps. Participants were also asked to list all livestock resource areas by name, identify their locations, and draw them on the map by hand (Figs. S3 & S4).

This activity was followed by broad discussions about animal movement characteristics of the village in relation to grazing and watering points, dips, resources containing minerals, livestock routes, sharing of grazing areas with wildlife and perceived disease risks. A structured list of questions guided the discussion to ensure consistency across villages and capture timeline of movements across seasons. All identified communal areas were categorised based on the absolute amount of use in wet, dry or both seasons, and ranked by the overall frequency of usage. However, given that the interpretation of seasons varies amongst villages, participants were asked to define the months corresponding to each season. For each type of resource area, participants described whether they were used by large or small herds or both, and the type of livestock species (i.e. cattle versus sheep and goats) that used them. These discussions were recorded using a digital field book and electronic voice recorder.

After each session, all key resource areas identified in the discussions were visited with the assistance of the village chairman. Their locations were validated, and their centres were geo-located using a hand-held global positioning system (GPS) device (Garmin eTrex® 10)

**Qualitative data analyses**

Using methods published elsewhere[3], qualitative data generated through the community participatory mapping discussions were extracted manually from the digital field notebook, voice recorder and hand drawn maps for each village and were recorded in text format in Excel. These data included: (1) all types of livestock resources used by livestock owners; (2) preferences (e.g. for grazing areas) and reasons for these; (3) herd size classification based on village perceptions, and the maximum number of livestock herds that could be moved as a single unit to resource areas; (4) predominant agricultural practices; (5) settlement types; (6) herding characteristics; and (7) season classifications and their corresponding months. Data were organised in tables that addressed specific objectives, for example, the types of resource areas where livestock herds were moved to, and season and herding duration at each resource area for each village.

## Wide-scale (trade-related) livestock movements

**Collection and processing of data on trade-related livestock movements.** Livestock movement permits (LMPs) were used to gather data on traded livestock. Information extracted from the permits included the village of the market where livestock were traded, village of destination, number of each species (cattle, sheep or goats) moved, and date and routes used, including major villages, towns and features such as hills or rivers encountered before the final destination. However, information on villages of origin is not typically recorded on LMPs. To capture these details, a field team member was stationed at the permit issuing point for each market to retrieve slips (local authorisation permits) containing information on livestock owner and village of origin which was then matched to LMPs. This was only possible for Serengeti District due to the availability of a dedicated field team member on every market day for the entire study period (January – December 2017). For the other districts, origin details were captured by a field assistant at each market who recorded village and number of all livestock traded on the market day by questioning owners of sold animals at movement permit issuing points. This information could only be gathered twice in the study period. Although it was not possible to match each origin data point to the exact destination, we were able to estimate the number of traded livestock that originated from each village or town. To augment this information, local livestock field officers, who are responsible for issuing permits in the district, were interviewed to identify major livestock catchment areas for each market. A short questionnaire was also administered to ten randomly selected livestock sellers and buyers to ascertain the origin of their livestock.

For each traded livestock species, data were aggregated at village level including primary origin, market at which they were sold, date, destination (village or town) and routes. To verify the completeness of the movement data extracted, the number of traded livestock obtained from the permit booklets were compared with government revenue reports that contained details of traded livestock for each district in the same year. To check for any inconsistencies, data cleaning was

performed in the R programming language version 3.6.1 [3], using dplyr version 0.8.3 [4] and reshape2 version 0.8.8 [5] packages.

**Data on human and livestock populations**. Information on human population size for each rural or urban location where livestock were traded, originated or headed to was extracted from the 2012 Tanzanian census report [1]. Livestock population numbers were obtained from government livestock branding reports (unpublished). In a few cases where permits indicated a town as the origin or destination, the mean human and livestock populations of the town was used.

## Supplementary Tables

**Table S1.** Themes and keywords from data that were collected during community-level participatory mapping activities to generate information on livestock movements. Data were recorded using a digital field notebook, a voice recorder and maps hand drawn by study participants. These were extracted manually and coded

| Herding parameter | Category | Key word extracted |
|---|---|---|
| Type of resource areas | Grazing or pasture | Grazing area, rangeland, pasture, crop waste, riverbanks, swamps, reserve areas, fodder banks |
| | Watering location | Rivers, dams, swamps, pools, distribution point, lakes, bore holes, wells |
| | Dipping locations | Dips, dipping house, dipping point. |
| | Salting points | Salt licks, river shores, salt blocks |
| Preferences | Pasture and water | Large grazing land, water point proximity, easy access, proximity to settlement area, proximity to salt points, all season pasture and water availability, |
| | Dip and Salt | Proximity (~5km or less), costs of dipping and salt cakes |
| Community perception/ classification of herd size | Large, medium, small | Agropastoral: large (>90), medium (89-30), small (<30)<br>Pastoral: large (>150), medium (149 – 90), small (<90) |
| Agricultural practices and livestock husbandry | Livestock, livestock-crops mixed systems | Cattle only, sheep only, goats only, sheep and goats, cattle and goats, cattle and sheep and goats, crops only and livestock and crops |
| Settlement types | Permanent and temporary | Fixed household, flexible household relocations, monthly household relocations, seasonal resettlement, bi-seasonal resettlement |

**Table S2.** Definitions of common network parameters and other terms used in the study. These refer to all types of networks we built to illustrate village connectivity and movement of traded livestock: (1) directed and bipartite networks, whereby nodes were resource areas and villages, and edges represented linkages between them, (2) undirected weighted networks, whereby nodes were villages and edge weights represented the shared resources between two villages and the frequency of use, and (3) directed weighted networks, whereby nodes were villages of livestock origin or destination, or market locations, while the edge weights were a function of the number of livestock traded between villages.

| Network parameter | Definition |
|---|---|
| Degree | The number of connections to or from a node (village) in a defined period (e.g. day, month or year). The bipartite network has directed edges (village to resource areas) which indicate their in-and-out degree. The single-node (village to village) network was undirected so there were no specifications for in-degree (contacts to a village) or out-degree (contacts from a village). The more the number of edges between connected villages, the higher the degree or contacts for the villages. |
| In-degree and out-degree | Using simplified definitions in our study context, a village weighted in-degree was the volume of livestock that the village received from other villages, while out-degree was the volume of livestock that left the village to other villages. |
| Betweenness | The term is used to describe the frequency by which a node falls between pairs of other nodes on the shortest path that connects them [6]. In our study, the weighted village betweenness describes the extent to which a village occupies positions in the network that influence the connections of others [7]. As a measure of centrality, node betweenness can be used to evaluate its ability to exert control or flow within a network. Villages with high betweenness were broadly considered ‘hub’ villages. Betweenness values range from 0 (least) to 1 (highest) |
| Eigenvector centrality | The term indirectly measures centrality determined by the centrality scores of the nodes to which the node of interest is connected to [7]. In our study, the eigenvector centrality value was defined loosely as the extent of how connected a village was to well-connected neighbours. It approximates the importance of each village in the network by assuming that each village centrality is the sum of the centrality values of the villages that it is connected to. A high eigenvector score means that a village is connected to many villages, which have high scores themselves. |

| | |
|---|---|
| Density | Number of edges in the observed network relative to the total number of possible edges in a completely connected network. |
| Village/inter-village connection | Contacts of at least one herd from one village with herd(s) from other village(s) at any resource area or market, i.e. when a herd visits a resource area of another village or when traded livestock are moved between two villages. This applies to both directed and undirected networks |
| Village contact (Unweighted degree) | Unique contacts between villages in a simplified network. It refers to the number of village(s) a village is connected to at least once in a defined period: day, week, month or year. |
| Connected components | A connected component of an undirected graph is a maximal set of nodes such that each pair of nodes is connected by a path. This also shows the partitions in the network. A component value of 1 will suggest all pairs of nodes were connected by a path in the network. |

**Table S3.** Variables included in the gravity models of livestock trade in the Mara region, northern Tanzania. Logit ($P_{ij}$) is the probability of movement (model 1) and Log ($\mathcal{M}_{ij}$) is the number of livestock movements from origin to destination village or town (model 2). $P_i$ and $P_j$ are the population of origin and destination. $D_{ij}$ is the distance between origin and destination. The equation includes also predictor variables ($X_k$) for origin or destination villages and their associated coefficients $\gamma_k$ are random effects for source and destination village. The initial model equation was written as follows:

$$\mathbf{logit}\,(\mathbf{P_{ij}}) = \boldsymbol{\beta_0} + \boldsymbol{\beta_1}\mathbf{P_i} + \boldsymbol{\beta_2}\mathbf{P_j} + \boldsymbol{\beta_3}\mathbf{D_{ij}} + \sum_{k=1}^{8}\boldsymbol{\gamma_k}\,\mathbf{X_k} + \mathbf{h_i} + \mathbf{h_j} \qquad \textbf{Model 1}$$

$$\mathbf{log}(\mathcal{M}_{ij}) = \boldsymbol{\beta_0} + \boldsymbol{\beta_1}\mathbf{P_i} + \boldsymbol{\beta_2}\mathbf{P_j} + \boldsymbol{\beta_3}\mathbf{D_{ij}} + \sum_{k=1}^{8}\boldsymbol{\gamma_k}\,\mathbf{X_k} + \mathbf{h_i} + \mathbf{h_j} \qquad \textbf{Model 2}$$

| Model parameter | | Coefficient type | |
|---|---|---|---|
| $D_{ij}$ | Pairwise distance of village of origin and destination (per standard deviation of the distribution) | Fixed effect | $\beta_3$ |
| $P_i$ | Livestock population number at origin (per standard deviation of the distribution) | Fixed effect | $\beta_1$ |
| $P_j$ | Livestock population number at destination (per standard deviation of the distribution) | Fixed effect | $\beta_2$ |
| $X_1$ | Human population number at origin (per standard deviation of the distribution) | Fixed effect | $\gamma_1$ |
| $X_2$ | Human population number at destination (per standard deviation of the distribution) | Fixed effect | $\gamma_2$ |
| $X_3$ | Market presence at origin (Yes/No) | Fixed effect | $\gamma_3$ |
| $X_4$ | Market presence at destination (Yes/No) | Fixed effect | $\gamma_4$ |
| $X_5$ | Origin was urban village/town (Yes/No) | Fixed effect | $\gamma_5$ |
| $X_6$ | Destination was urban village/town (Yes/No) | Fixed effect | $\gamma_6$ |
| $X_7$ | Abattoir at destination (Yes/No) | Fixed effect | $\gamma_7$ |
| $X_8$ | Market presence at origin and destination (Yes/No) | Fixed effect | $\gamma_8$ |
| - | Origin village name | Random effect | $h_i$ |
| - | Destination village name | Random effect | $h_j$ |

**Table S4**. Summary of key livestock herding characteristics practised by pastoral and agropastoral villages in the study area in northern Tanzania.

| Herding parameter | Herding pattern | |
|---|---|---|
| | **Agropastoral** | **Pastoral** |
| Land use plan | Not frequently used and difficult to enforce. Only 10/97 villages indicated that they had allocated permanent areas in the village for livestock grazing, cropping and human settlements. | Very few villages (2/46) have dedicated areas for grazing and human settlements. |
| Settlement, re-settlements, and camping | All village households had one permanent settlement. Crop- and livestock-related activities (e.g. grazing) were performed daily out in the field. Herds were returned back to settlements every day. Most households in all villages engaged in cropping more than livestock keeping. A few livestock-owning households (in 15 out of 97 villages) engaged in camping whereby cattle were moved to neighbouring villages for about 6-8 weeks until the beginning of the wet season. Most households grazed small and large ruminant herds of all sizes together. Few livestock-keeping households with large herds (>100 cattle) travelled longer distances to large grazing fields to avoid crop cultivated areas. | The majority of village households (40/46) had one permanent and several temporary settlements. All livestock in the herds that were seasonally relocated were returned back to permanent settlements once or twice in a season depending on climatic conditions and rainfall patterns. Settlement and resettlement around rangelands were mostly influenced by forage levels, predation and risk of malignant catarrhal fever, a disease of livestock associated with wildebeest calving. Broadly, most herds were moved back to permanent settlements at the end of the wet season, where they would stay for 2-3 months before relocating to temporary settlements. About 6 villages considered themselves to be absolute pastoralists without permanent settlements and no crop activities. |

| | | |
|---|---|---|
| Herding | Herding decisions were not influenced by the need to access mineral salts or water. | Mixed livestock types were kept in the same household and were grazed together. However, 10% of livestock, including pregnant, young and sick animals were usually left at permanent settlements during long-distance migrations. Some herders reported moving at least one livestock herd between the temporarily and permanently located herds every 4 -12 weeks depending on the location of the camping site and precipitation levels. Common community perceptions are that mineral salt is vital for reproduction therefore herding decisions are mostly influenced by a need for mineral salt supplements or water during extreme dry season. |

**Table S5**. Properties of livestock movement networks (see also Supplementary Figures S7-S9) in agropastoral and pastoral production systems at each of the four major resource areas, grazing, watering, salting and dipping, identified through participatory mapping in two districts of northern Tanzania, Serengeti (agropastoral) and Ngorongoro (pastoral). Nodes are villages, while edge weights are a function of the number of shared resources between villages - an indication of the strength of contact.

| Network statistics | Agropastoral | | | | Pastoral | | | |
|---|---|---|---|---|---|---|---|---|
| | Grazing | Water | Salt | Dip | Grazing | Water | Salt | Dip |
| Size | | | | | | | | |
| node | 97 | 91 | 51 | 54 | 46 | 45 | 41 | 5 |
| edge | 5654 | 2880 | 1400 | 1715 | 9048 | 10901 | 5525 | 52 |
| Betweenness | | | | | | | | |
| min | 0.00 | 0.00 | 0.00 | 0.00 | 0.00 | 0.00 | 0.00 | 0.00 |
| median | 0.01 | 0.01 | 0.00 | 0.00 | 0.01 | 0.01 | 0.00 | 0.00 |
| mean | 0.04 | 0.03 | 0.00 | 0.01 | 0.04 | 0.04 | 0.03 | 0.03 |
| max | 0.23 | 0.29 | 0.06 | 0.17 | 0.29 | 0.46 | 0.25 | 0.16 |
| Eigenvector | | | | | | | | |
| min | 0.00 | 0.00 | 0.00 | 0.00 | 0.01 | 0.00 | 0.00 | 0.00 |
| median | 0.10 | 0.01 | 0.00 | 0.00 | 0.49 | 0.13 | 0.37 | 0.16 |
| mean | 0.21 | 0.05 | 0.10 | 0.15 | 0.45 | 0.24 | 0.33 | 0.43 |
| max | 1.00 | 1.00 | 1.00 | 1.00 | 1.00 | 1.00 | 1.00 | 1.00 |
| Village contact (unweighted degree) | | | | | | | | |
| min | 1 | 1 | 1 | 1 | 2 | 3 | 1 | 1 |
| median | 7 | 5 | 3 | 3 | 15 | 15 | 14 | 2 |
| mean | 7 | 5 | 3 | 4 | 16 | 16 | 12 | 2 |
| max | 19 | 19 | 8 | 13 | 28 | 30 | 23 | 2 |
| Density | 0.08 | 0.06 | 0.06 | 0.07 | 0.36 | 0.37 | 0.31 | 0.40 |

**Table S6**. Properties of livestock movement networks for each production system across seasons. Nodes are villages, while edges are the number of contacts among connected villages including a drought year we captured in the pastoral setting. All centrality measures are weighted.

| Network statistics | Agropastoral | | | Pastoral | | | |
|---|---|---|---|---|---|---|---|
| | Wet | Dry | Year | Wet | Dry | Drought | Year |
| Size | | | | | | | |
| node | 94 | 96 | 97 | 46 | 44 | 45 | 46 |
| edge | 2349 | 4487 | 11906 | 6256 | 4000 | 6656 | 23128 |
| Betweenness | | | | | | | |
| min | 0.00 | 0.00 | 0.00 | 0.00 | 0.00 | 0.00 | 0.00 |
| median | 0.02 | 0.01 | 0.01 | 0.01 | 0.02 | 0.01 | 0.01 |
| mean | 0.03 | 0.03 | 0.03 | 0.04 | 0.05 | 0.03 | 0.04 |
| max | 0.28 | 0.30 | 0.22 | 0.50 | 0.36 | 0.22 | 0.35 |
| Eigenvector | | | | | | | |
| min | 0.00 | 0.00 | 0.00 | 0.00 | 0.00 | 0.00 | 0.00 |
| median | 0.00 | 0.07 | 0.02 | 0.39 | 0.05 | 0.09 | 0.39 |
| mean | 0.08 | 0.17 | 0.14 | 0.32 | 0.17 | 0.22 | 0.36 |
| max | 1.00 | 1.00 | 1.00 | 1.00 | 1.00 | 1.00 | 1.00 |
| Village contact (unweighted degree) | | | | | | | |
| min | 1 | 1 | 7 | 1 | 3 | 4 | 3 |
| median | 5 | 8 | 8 | 18 | 11 | 17 | 20 |
| mean | 5 | 8 | 9 | 15 | 13 | 17 | 20 |
| max | 9 | 22 | 22 | 28 | 24 | 31 | 33 |
| Density | 0.05 | 0.09 | 0.09 | 0.33 | 0.30 | 0.39 | 0.45 |
| Proportion of connections contributed by top 20% high degree villages | 0.29 | 0.35 | 0.49 | 0.35 | 0.32 | 0.45 | 0.47 |

**Table S7**. The number of livestock that were traded in the study districts in the year 2017. DC and TC are District Council and Town council, respectively.

| District | Livestock category | | |
|---|---|---|---|
| | Cattle | Sheep | Goats |
| Serengeti | 18486 | 2227 | 3715 |
| Tarime | 7276 | 1031 | 1379 |
| Bunda DC | 6502 | 1028 | 5105 |
| Bunda TC | 3954 | 753 | 2264 |
| Butiama | 9729 | 442 | 3509 |

**Table S8**. District of origin of traded livestock in the study area. DC = District Council, TC = Town Council.

| District of market location | District of origin | | | | | | |
|---|---|---|---|---|---|---|---|
| | Serengeti | Tarime | Bunda DC | Bunda TC | Butiama | Rorya | Musoma |
| Serengeti | 21,879 | 76 | 201 | 0 | 344 | 0 | 0 |
| Tarime | 2,409 | 3,311 | 1,100 | 91 | 772 | 22 | 0 |
| Bunda DC | 1,300 | 67 | 12,037 | 1,028 | 26 | 0 | 0 |
| Bunda TC | 0 | 0 | 1,772 | 5,001 | 400 | 0 | 0 |
| Butiama | 2,166 | 31 | 77 | 9 | 12,985 | 0 | 6 |

**Table S9**. Significant explanatory variables from the final binomial generalised linear mixed model of probability of traded livestock movements between village pairs. The continuous variable (pairwise distance between origin and destination) was scaled to mean zero and one standard deviation. CI denotes Confidence Interval.

| | Estimate (95% CI) | P value | Odds Ratio (95% CI) |
|---|---|---|---|
| Pairwise distance between origin and destination (per standard deviation of the distribution) | -0.99 (-1.20 - 0.79) | <10^-6 | 0.37 (0.23 – 0.49) |
| Market presence at origin (Yes vs No) | 1.37 (1.17 – 1.57) | <10^-6 | 3.96 (3.25– 4.74) |
| Market presence at destination (Yes vs No) | 1.87 (1.67 – 2.09) | <10^-7 | 6.53 (5.31-8.00) |

## Supplementary Box

**Box S1.** Details of the four types of key resource areas that drove connectivity between villages in both agropastoral and pastoral systems.

**(a)** *Grazing areas*: Movements to grazing areas occurred daily during the wet and dry seasons. Study participants reported that some villages have specific areas allocated to grazing, while others have open grazing plans where livestock are allowed to graze on pastures without restrictions. In agropastoral settings, a village was connected to seven (unweighted mean degree) other villages through 9.6 (116/12) links (i.e. weighted mean degree, which also indicate mean movement events where livestock herds from a village encountered herds from other villages) across several grazing areas in a month (Table S5). In pastoral settings, between-village connections at grazing locations were almost twice as numerous as in agropastoral systems despite the network having half the number of nodes (Table S5 and Figs. S7 & S8).

**(b)** *Watering points*: Similar to grazing areas, study participants reported daily movements of livestock to water sources across seasons. However, in extremely dry periods, pasture is typically prioritised over water, hence livestock movements to water sources occur every other day. Each agropastoral village was connected to five others through watering points (Table S5 and Fig. S7), apart from three that had no contacts with other villages in a whole year. In the agropastoral settings, the links at watering points were fewer compared to grazing, while the opposite was observed in the pastoral settings (Table S5 and Figs. S7 & S8).

**(c)** *Salting points*: Movements to salt licks, used as dietary supplements (particularly for cattle), occurred weekly and more often involved pastoralists compared to agropastoralists (Table S5 and Figs. S7 & S9). A village was connected to three (unweighted degree, agropastoral) and 12 (unweighted degree, pastoral) other villages through this resource area.

**(d)** *Dipping points*: Routine (once or twice a month) dipping was practised in more than 56% (54/97) of agropastoral villages, but it was less common (11%, 5/46) in pastoral systems (Table S5 and Figs. S7 & S9). Consistent with this, a village was connected to 3.5 (unweighted mean degree, agropastoral) and 1.6 (unweighted mean degree, pastoral) other villages through livestock dips (Table S5).

# Supplementary Figures

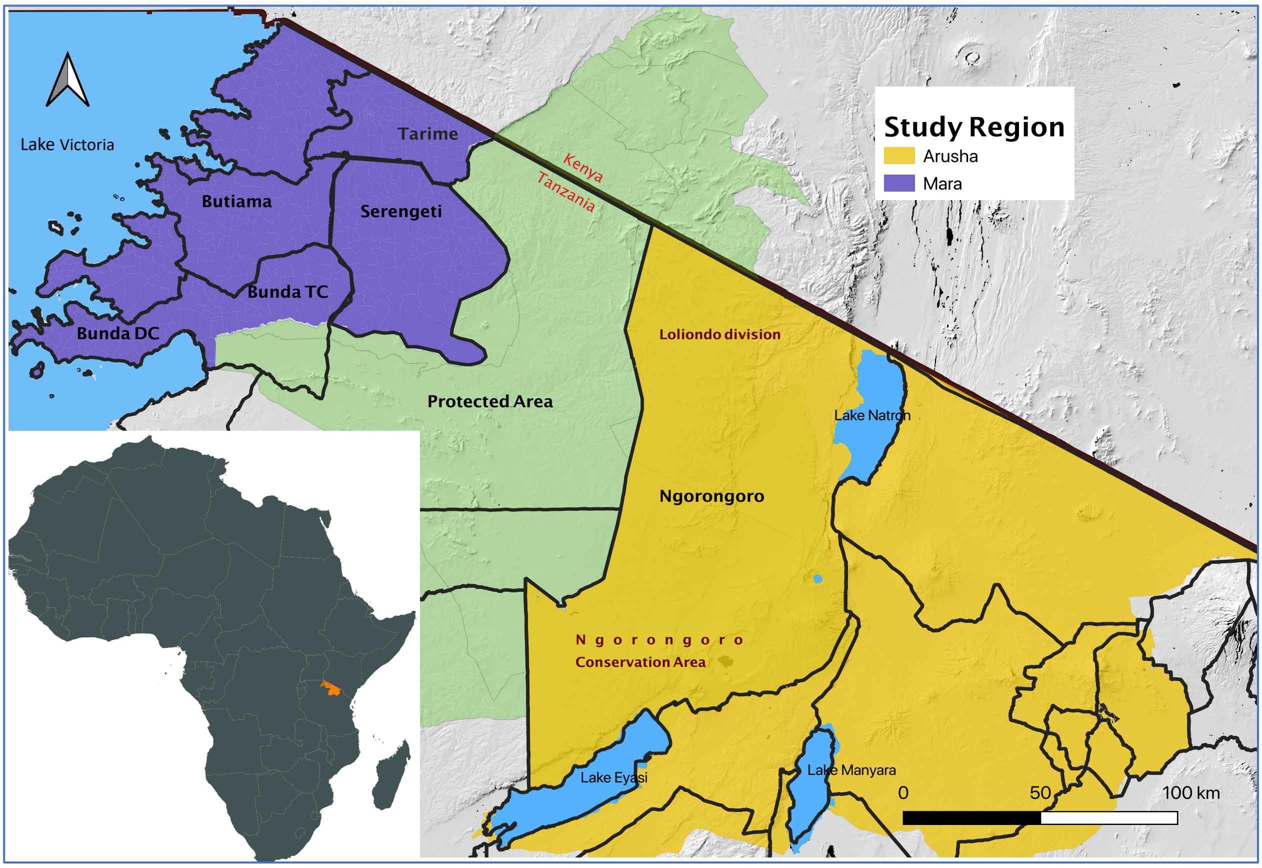


**Figure S1**. A map of the study area in northern Tanzania and its location within the map of Africa (left). The districts of focus to generate data on local-level livestock movements were Serengeti District (agropastoral) to the west of the Serengeti Ecosystem and Ngorongoro District (pastoral) to the east. Wide-scale (trade-related) movements were investigated in four agropastoral districts, Serengeti, Bunda, Butiama and Tarime, to the west of the Serengeti Ecosystem. The shapefiles were based on 2012 Tanzania census, obtained from Tanzania National Bureau of Statistic. The map was developed in Quantum geographical information system (QGIS), version 3.18 [2].

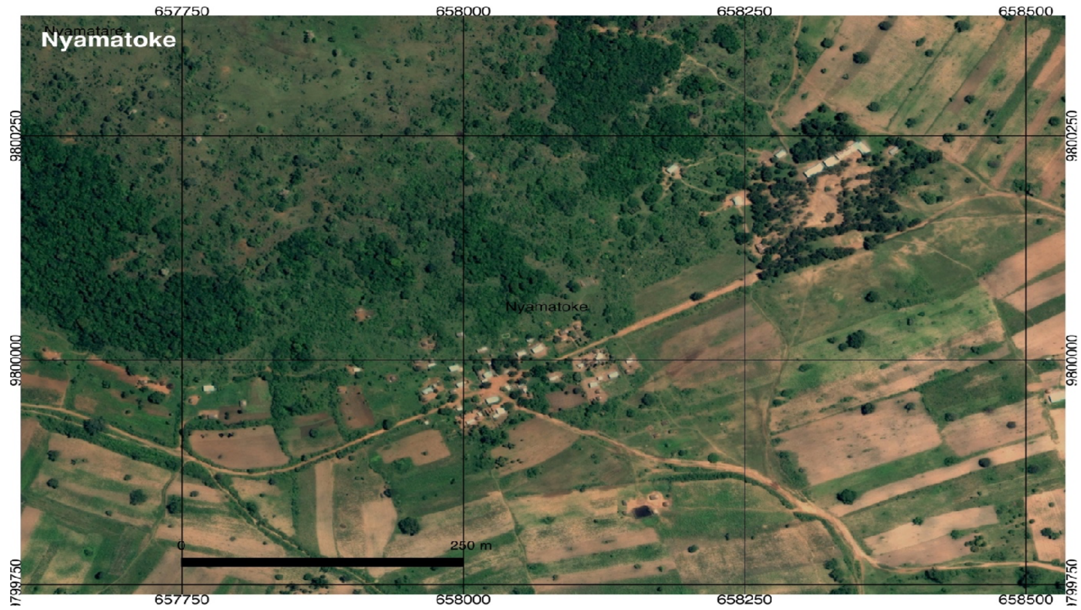


**Figure S2**. An example of a Google Earth digital map with built-in coordinate system at 1:2500 scale that was used to map livestock movements to key livestock resources in an agropastoral village (Nyamatoke). Topographical features shown include settlements, hills, roads, crop lands, grazing reserves and utility centres. The map was developed in Quantum geographical information system (QGIS), version 3.18, using in-built Google Satellite Open Maps, in Quick Map Services Plug In [2].

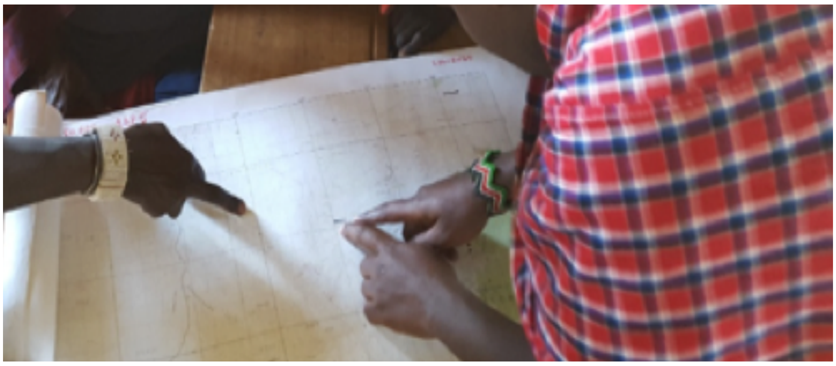

**Figure S3**. Picture showing participants of a participatory mapping session in a pastoral community in Ngorongoro District, northern Tanzania, identifying key resource areas (e.g. rangeland and water source) and navigating livestock routes on printed base maps at 1:50000 scale.

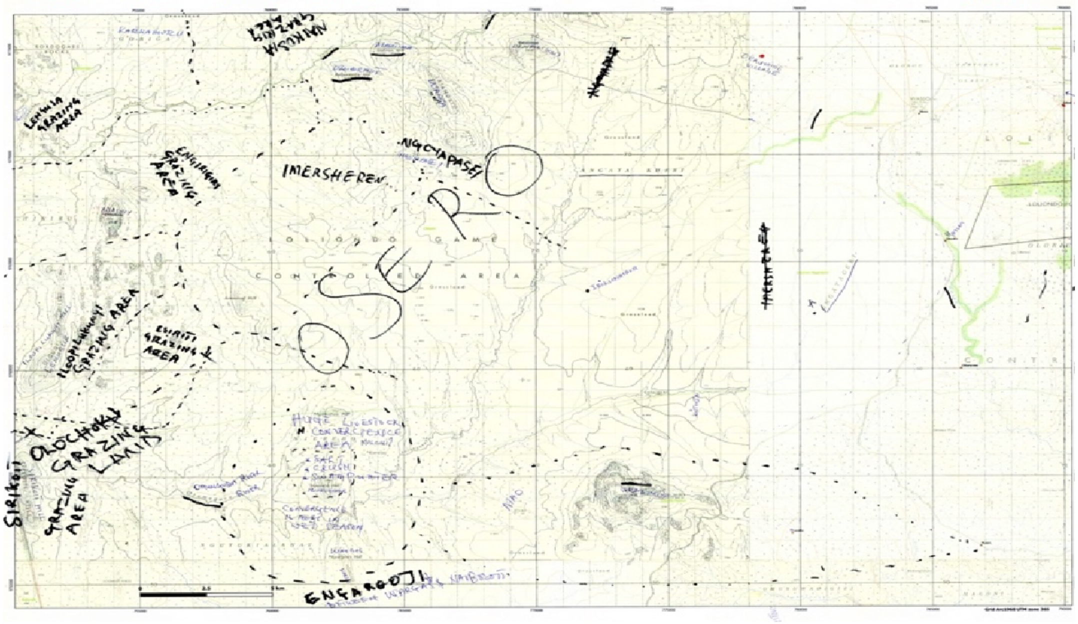

**Figure S4**. An example of a hand-drawn map (1:50000 scale) produce during a participatory mapping session showing different sections of the largest rangeland in Loliondo, Ngorongoro District, northern Tanzania. The shapefiles on the base map were based on 2012 Tanzania census, obtained from Tanzania National Bureau of Statistic. The map was developed in Quantum geographical information system (QGIS), version 3.18[2].

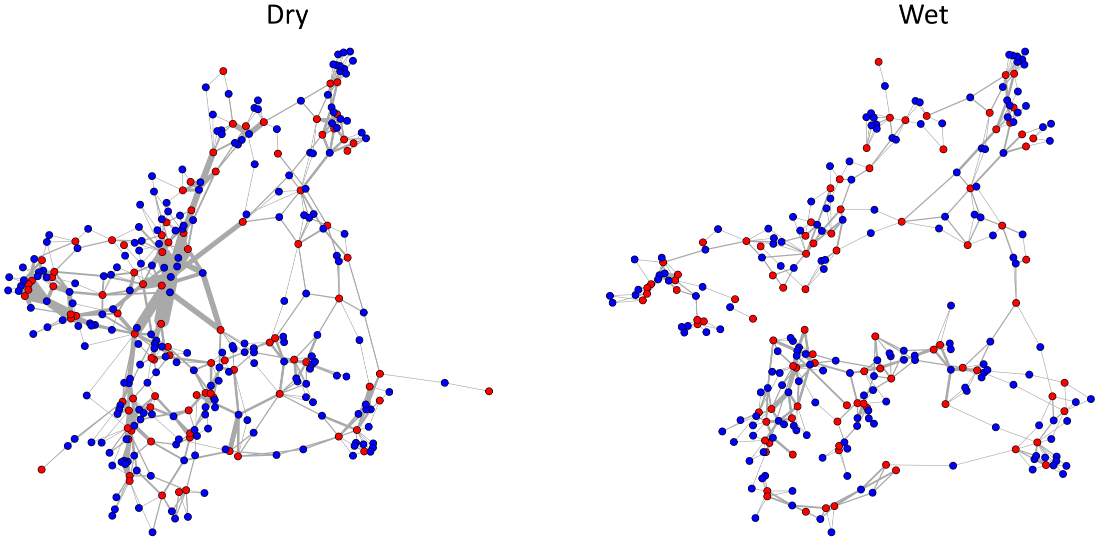


**Figure S5**. A weighted topological directed network showing seasonality of connected villages in the Serengeti District through shared resource areas during a month in the wet and dry seasons. Gray lines are weighted edges. Red dots are villages and blue dots are shared resource areas.

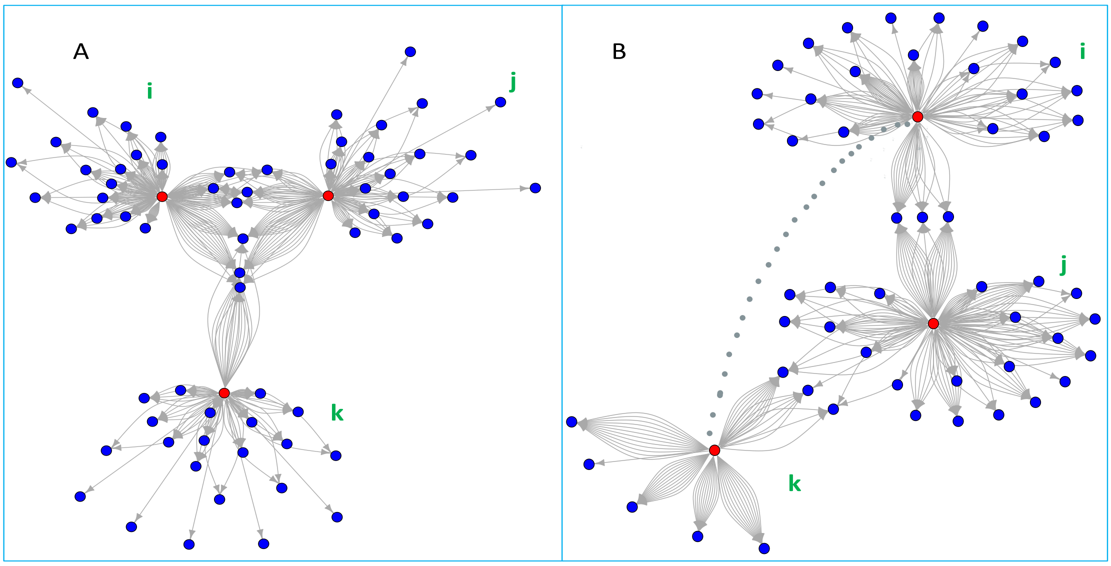


**Figure S6**. A schematic network of connectivity amongst villages (red nodes) through resource areas (blue nodes) shared by livestock herds in  agropastoral and pastoral systems of northern Tanzania investigated in this study. The letters “i”, “j” and “k” refer to villages in the topological network. Panel A shows connectivity between the three villages through direct access to shared resource areas (i.e. instances where herds converge into the same resource areas located within the three villages). In panel B, connectivity is through direct access to shared resource areas between villages “i” and “j”, and “k” and “j”, while villages “i” and “k” are connected only indirectly (dotted line) (i.e. connectivity is being mediated by a third village).

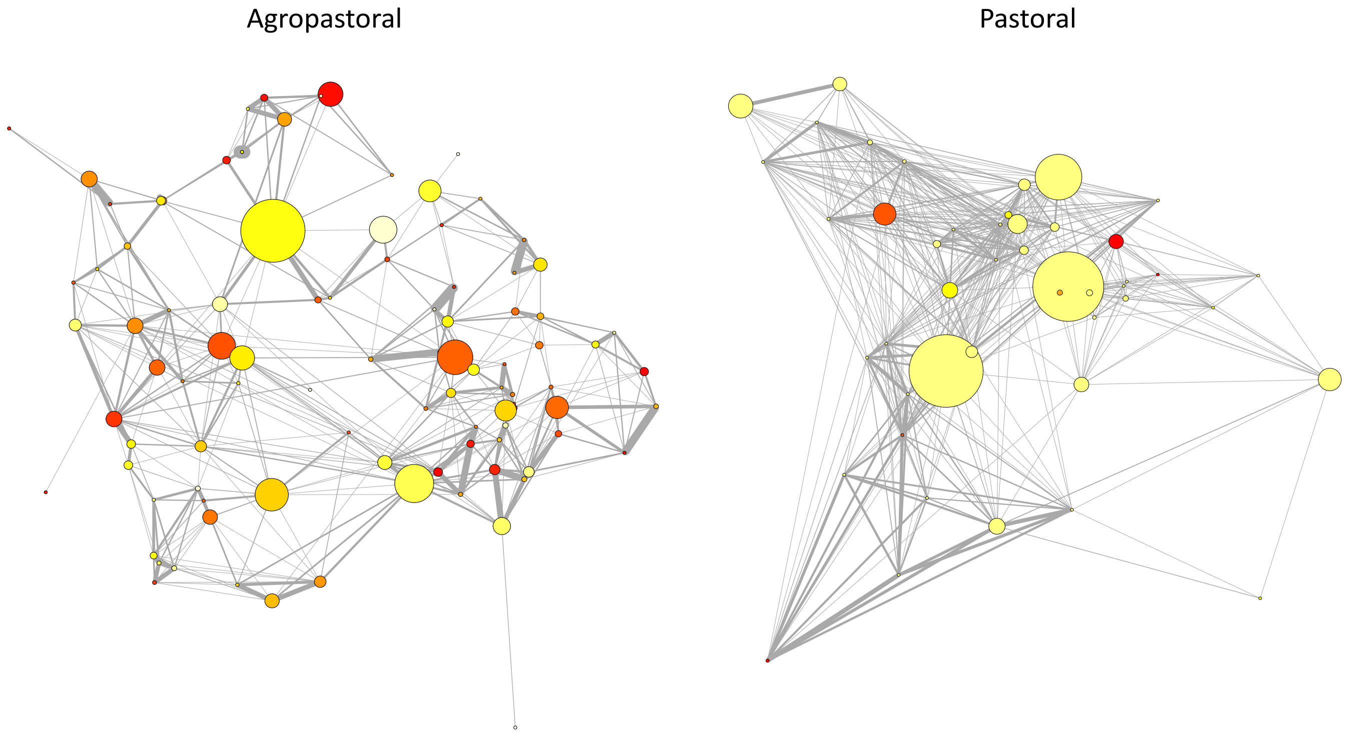


**Figure S7**. Spatial livestock movement networks of village connectivity in agropastoral and pastoral systems of northern Tanzania investigated in this study. The nodes (circles) are villages in their geographical position. The grey weighted edges represent shared resource areas (i.e. grazing, watering, salting and dipping areas) among connected villages. The weighted edges represent the centrality measure degree, i.e. the total number of contacts of a village relative to other villages. The length of each edge is the proportional distance between connected village centres. The node size is proportional to the network centrality measure betweenness for the village, i.e. the ability of a village to occupy positions in the network that influence the connections of others. Node colours refer to eigenvector centrality values, which quantify the extent to which a village with high degree was in contact with other high-degree villages. The colour red indicates the highest eigenvector centrality value.

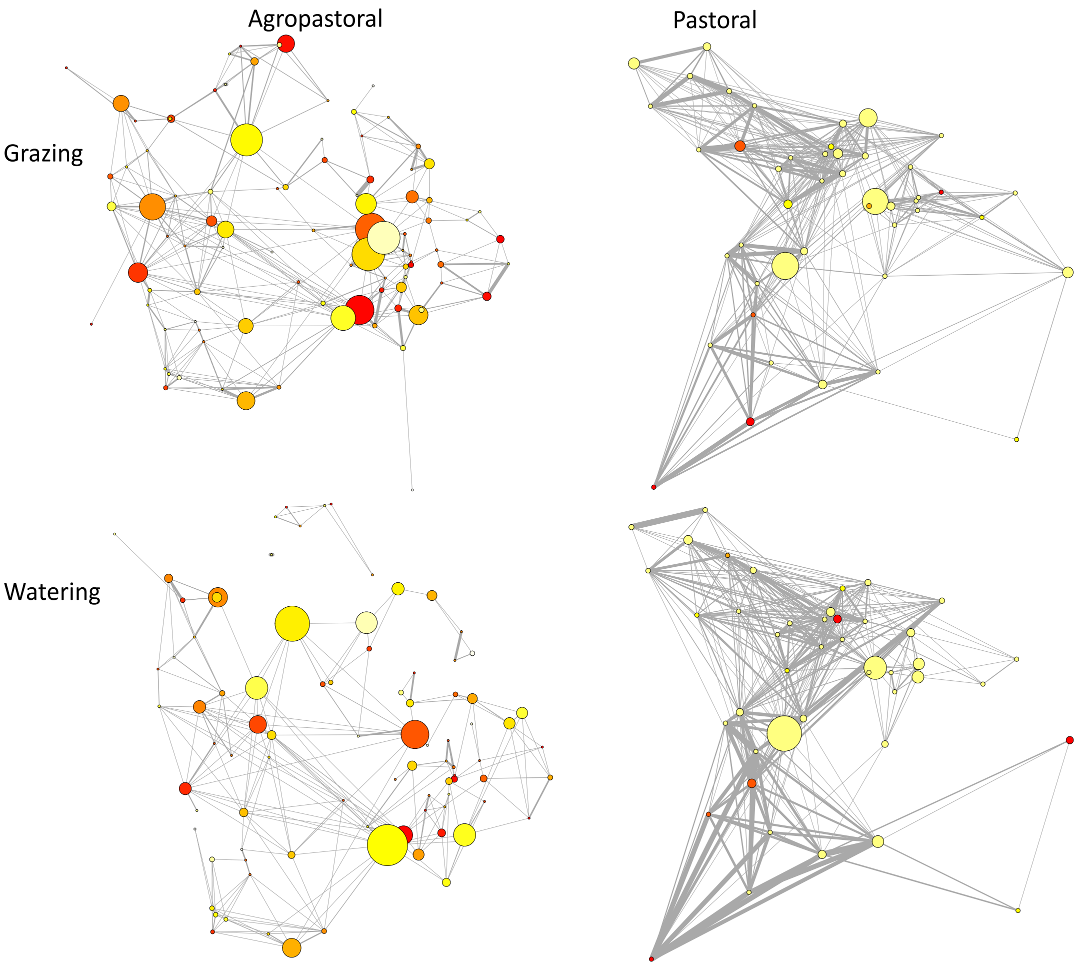


**Figure S8**. Networks of village livestock connectivity at grazing and watering locations across management settings, agropastoral and pastoral, of northern Tanzania investigated in this study.The nodes (circles) are villages in their geographical position, node sizes are proportional to the network centrality measure (betweenness) for the village, and node colours correspond to the centrality measure centrality value. The grey edges represent shared resource areas among connected villages, the number of edges is the centrality measure “degree”, while the length of each edge is the proportional distance between connected village centres. Degree measures the total number of contacts of a village relative to other villages, betweenness describes the ability of a village to occupy positions in the network that influence the connections of others and eigenvector value quantifies the extent by which a village with high degree was in contact with other high degree villages.

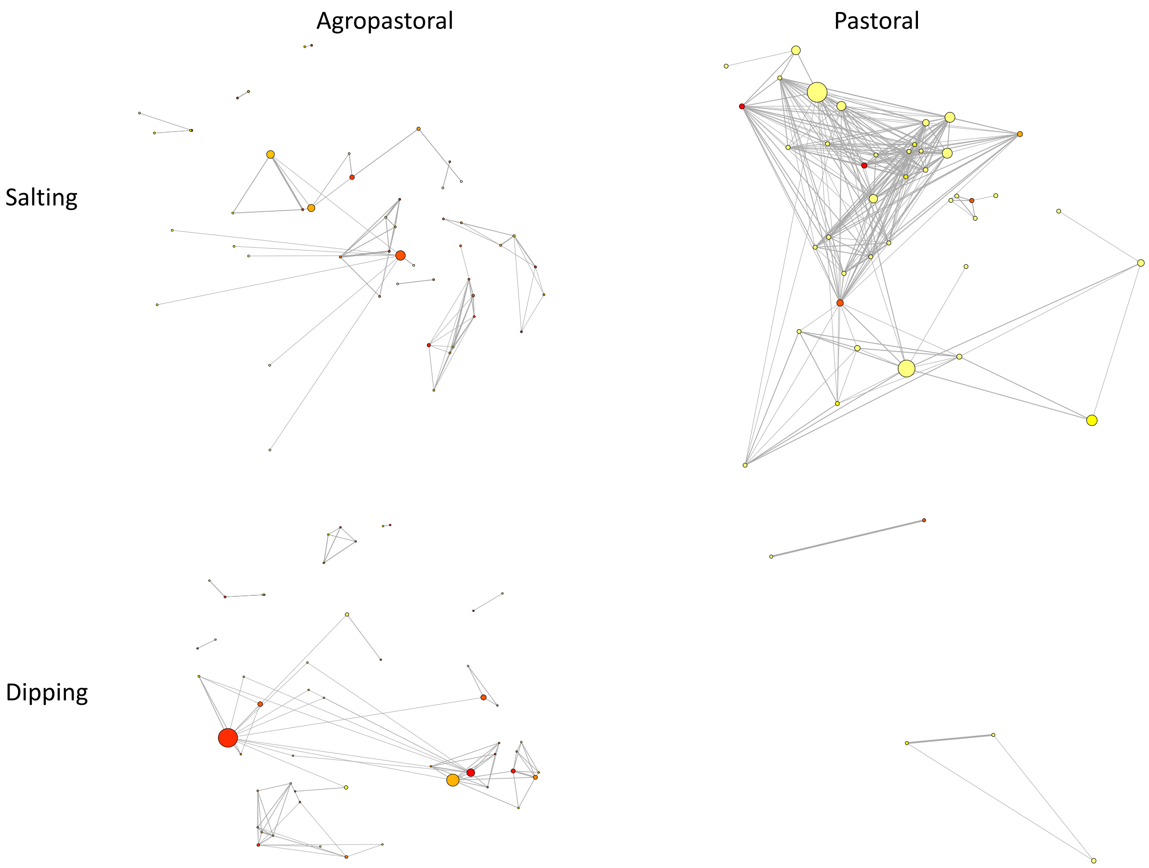


**Figure S9**. Networks of village livestock connectivity at salting and dipping pionts across management settings, agropastoral and pastoral, of northern Tanzania investigated in this study. The nodes (circles) are villages in their geographical position. Node sizes are proportional to the network centrality measure (betweenness) for the village and the node colour is the eigenvector centrality value. The grey edges represent shared resource areas among connected villages. The number of edges corresponds to the centrality measure degree, while the length of each edge is the proportional distance between connected village centres. Degree measures the total number of contacts of a village relative to other villages, betweenness describes the ability of a village to occupy positions in the network that influence the connections of others and eigenvector value quantifies the extent by which a village with high degree was in contact with other high-degree villages.

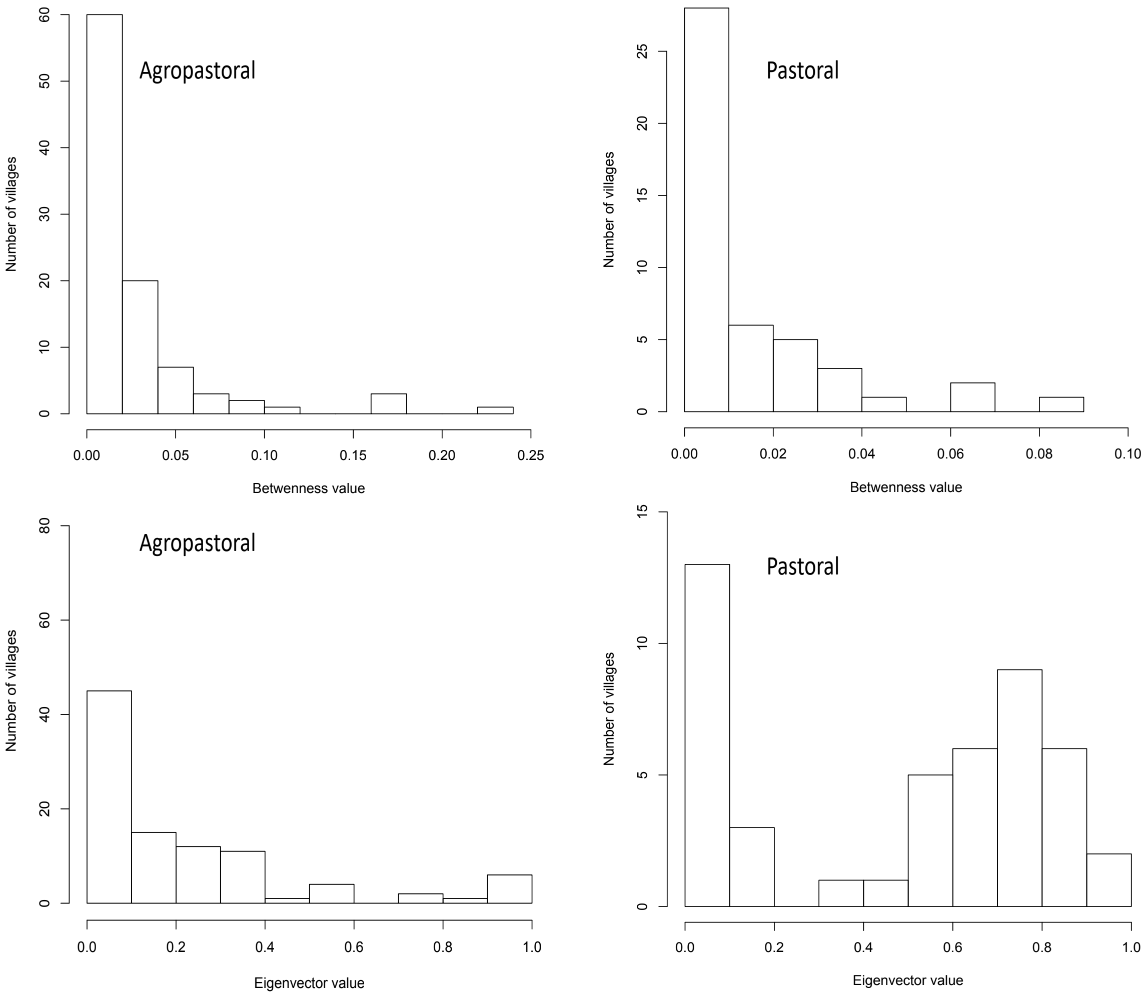


**Figure S10**. Distribution of normalised centrality betweeenness and eigenvector centrality values for agropastoral and pastoral networks generated from participatory mapping data in northern Tanzania. Few villages had relatively high centrality values suggesting their importance in the network. Betweenness describes the ability of a village to occupy positions in the network that influence the connections of others and eigenvector values quantifiy the extent by which a village with high degree is in contact with other high degree villages.

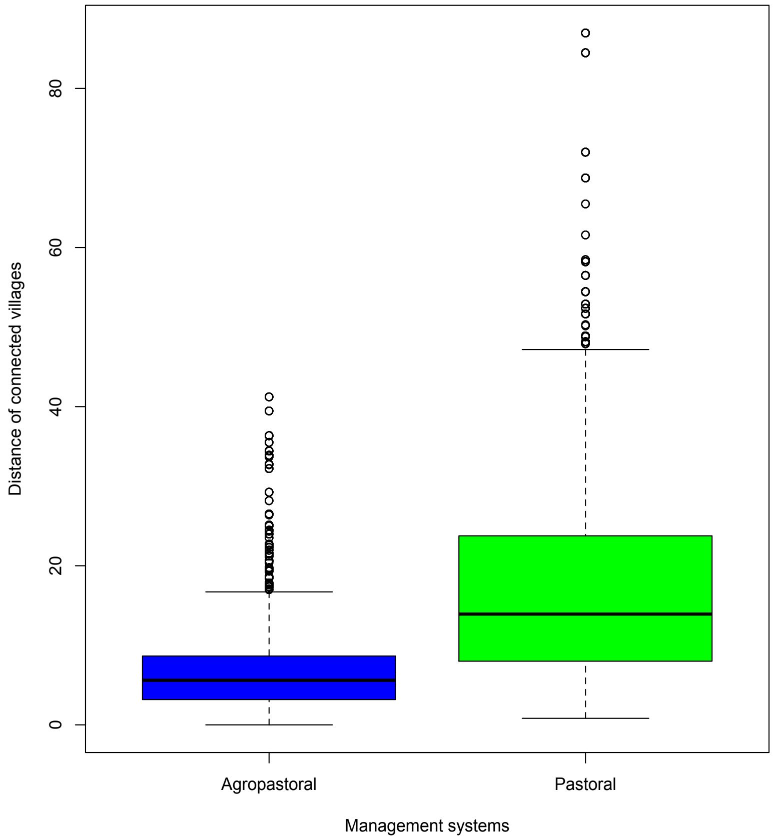


**Figure S11**. Distance (km) of connected villages across livestock management systems, agropastoral and pastoral, investigated in northern Tanzania.

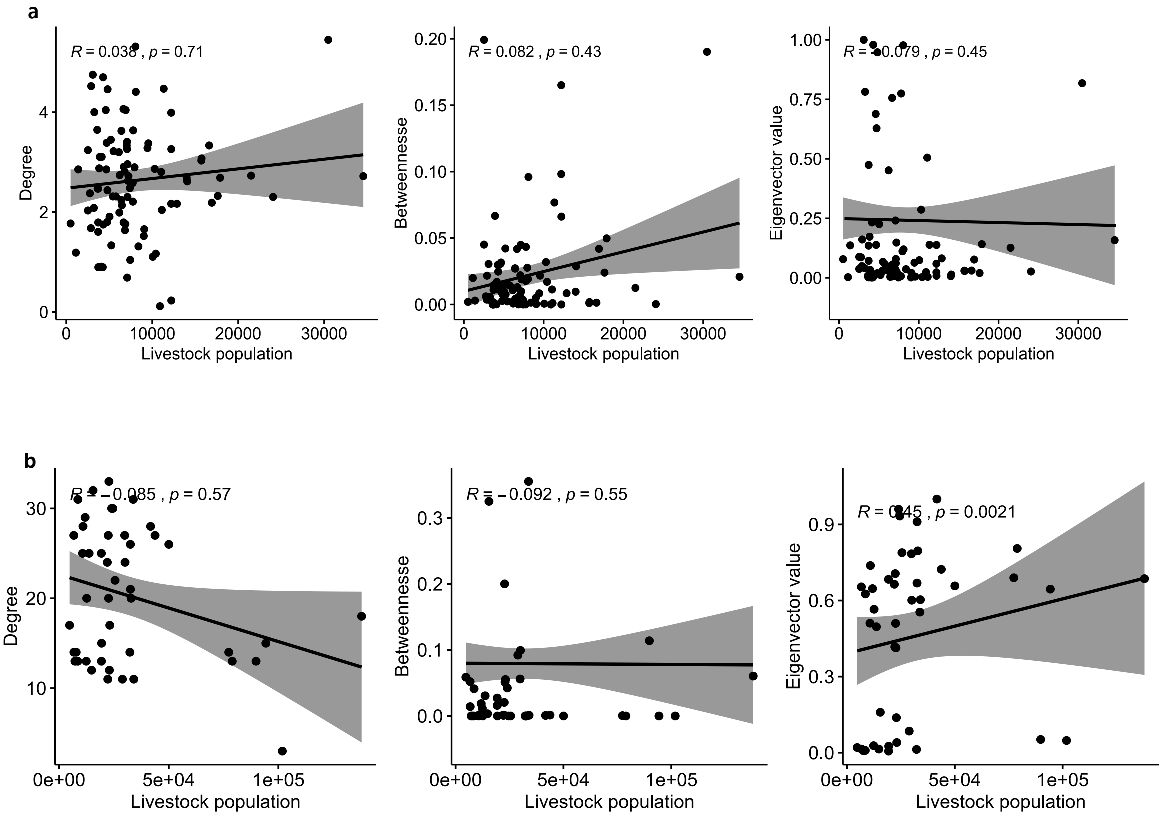

**Figure S12**. Spearman correlation test showing the relationship between village livestock populations (i.e cattle, goats and sheep) and the weighted and normalised network centrality measures in (a) agropastoral and (b) pastoral livestock management systems investigated in northern Tanzania as part of this study.

## References


1. Tanzania National Bureau of Statistics: Population and Housing Census. 2012. *https://www.nbs.go.tz/index.php/en/census-surveys/population-and-housing-census*
2. QGIS Development Team. Quantum Geographic Information System. Open Source Geospatial Foundation Project. 2020; 3.16. https://qgis.org/en/site/forusers/download.html.
3. R Core Team. R: A language and environment for statistical computing. R Foundation for Statistical Computing, Vienna, Austria. 2020. ## URL https://www.R-project.org/.
4. Wickham H, Averick M, Bryan J, Chang W, McGowan LD, François R, Grolemund G, Hayes A, Henry L, Hester J, Kuhn M, Pedersen TL, Miller E, Bache SM, Müller K, Ooms J, Robinson D, Seidel DP, Spinu V, Takahashi K, Vaughan D, Wilke C, Woo K, Y. H. "Welcome to the tidyverse". *J. Open Source Softw.* **4**, 1686 (2019).
5. Wickham, H. Reshaping Data with the reshape Package. *J. Stat. Softw.* **21**, 1–20 (2007).
6. Freeman, L. C. Centrality in social networks: conceptual clarification. *Soc. Networks* **1**, 215–239 (1979).
7. Wasserman, S. & Faus, K. *Social network analysis: methods and applications (Vol. 8).* (Cambridge University Press, 1994).
